# Tumor Cell-Autonomous Pro-Metastatic Activities of PD-L1 in Human Breast Cancer Are Mediated by PD-L1-S283 and Chemokine Axes

**DOI:** 10.3390/cancers14041042

**Published:** 2022-02-18

**Authors:** Nofar Erlichman, Tamir Baram, Tsipi Meshel, Dina Morein, Benny Da’adoosh, Adit Ben-Baruch

**Affiliations:** 1 The Shmunis School of Biomedicine and Cancer Research, George S. Wise Faculty of Life Sciences, Tel Aviv University, Tel Aviv 69978-01, Israel; nofarerlichman@gmail.com (N.E.); tamiros21@gmail.com (T.B.); tsipi.meshel@gmail.com (T.M.); dinamorein1@gmail.com (D.M.); 2 Blavatnik Center for Drug Discovery, Tel Aviv University, Tel Aviv 69978-01, Israel; daadoosh@tauex.tau.ac.il

**Keywords:** chemokines, CCR2, CCR5, CXCR1/2, luminal-A breast cancer, PD-1, PD-L1, triple-negative breast cancer (TNBC)

## Abstract

**Simple Summary:**

Triple-negative breast cancer (TNBC) is an aggressive disease that responds in a limited manner to immune checkpoint blockades targeting the PD-L1/PD-1 axis, suggesting that PD-L1 potentiates TNBC progression via pathways not related to immune suppression. We demonstrated that, in human breast cancer cells, PD-L1 expression increased in a cell-autonomous manner tumor cell growth, invasion and release of pro-metastatic factors; these activities were elevated by exposure to PD-1 and were markedly impaired in S283-mutated PD-L1-expressing cells. Invasion of WT-PD-L1-expressing TNBC cells depended on autocrine chemokine circuits, involving CXCR1/2, CCR2, CCR5 and their ligands. In T cell-deficient mice, WT-PD-L1 exhibited increased tumor growth and metastasis by TNBC cells, whereas S283A-PD-L1-expressing cells showed a very poor tumorigenic and metastatic profile. These findings on cell-autonomous and PD-1-induced pro-metastatic activities of PD-L1 in cancer cells suggest that treatments targeting PD-L1 could improve the efficacy of immune-targeting checkpoint inhibitors, e.g., anti-PD-1 or anti-CTLA-4 in TNBC.

**Abstract:**

Therapies targeting the PD-L1/PD-1 axis have recently been introduced to triple-negative breast cancer (TNBC) with limited efficacy, suggesting that this axis promotes tumor progression through mechanisms other than immune suppression. Here, we over-expressed WT-PD-L1 in human TNBC cells (express endogenous PD-L1) and in luminal-A breast cancer cells (no endogenous PD-L1 expression) and demonstrated that cell-autonomous PD-L1 activities lead to increased tumor cell growth, invasion and release of pro-metastatic factors (CXCL8, sICAM-1, GM-CSF). These activities were promoted by PD-1 and were inhibited by mutating S283 in PD-L1. Invasion of WT-PD-L1-cells required signaling by chemokine receptors CXCR1/2, CCR2 and CCR5 through autocrine circuits involving CXCL8, CCL2 and CCL5. Studies with T cell-deficient mice demonstrated that cell-autonomous WT-PD-L1 activities in TNBC cells increased tumor growth and metastasis compared to knock-out (KO)-PD-L1-cells, whereas S283A-PD-L1-expressing cells had minimal ability to form tumors and did not metastasize. Overall, our findings reveal autonomous and PD-1-induced tumor-promoting activities of PD-L1 that depend on S283 and on chemokine circuits. These results suggest that TNBC patients whose tumors express PD-L1 could benefit from therapies that prevent immune suppression by targeting PD-1/CTLA-4, alongside with antibodies to PD-L1, which would allow maximal impact by mainly targeting the cancer cells.

## 1. Introduction

Breast cancer is a heterogenous disease, in which different subtypes were identified based on the presence or lack of expression of three receptors: estrogen receptor α, progesterone receptor and HER2. The triple-negative subtype of breast cancer (TNBC; corresponding to the “basal” subtype of breast cancer in genomic analyses) accounts for 15–20% of the patients and is identified by the lack of all three receptors. As a result, TNBC patients cannot be treated by targeted therapies and, thus, the treatment of choice in TNBC is chemotherapy [1,2,3]. Whereas a considerable part of TNBC patients responds initially to chemotherapy, many of them eventually relapse [3,4,5]. Overall, TNBC is identified as a most aggressive disease, emphasizing the need to identify novel therapeutic modalities in this disease subtype.

Immunotherapies that use antibodies directed to inhibitory immune checkpoints—such as PD-L1, PD-1 and CTLA-4—have introduced a major breakthrough in cancer therapy, with relatively good impact on disease course, primarily in melanoma [6,7]. In breast cancer, it was suggested that treatment by immune checkpoint blockades (ICBs) may apply particularly to TNBC tumors because of their relatively high mutation rate, which is expected to generate neo-antigens [8,9,10,11]. Studies of inhibitory immune checkpoints in TNBC have addressed mainly the PD-L1/PD-1 axis; they demonstrated the expression of PD-L1 and PD-1 by cancer cells and immune cells in the tumor and analyzed their connection to patient survival. Determination of PD-L1 association with prognosis is partly hampered by the fact that the molecule is expressed by the cancer cells and by leukocytes at the tumor site, mainly macrophages. Some of the studies that were done in this direction have found that the PD-L1/PD-1 axis is connected to poor prognosis in TNBC. However, PD-L1 expression was, at times, associated with improved survival, primarily when high levels of tumor-infiltrating lymphocytes, which, by themselves, are connected to better prognosis in TNBC, were present in the tumors. This observation is presumed to reflect stronger immune activation and the dynamic nature of immune activities at the tumor site [11,12,13,14]. 

In view of these studies and the well-identified roles of the PD-L1/PD-1 axis in inhibiting immune surveillance, several clinical trials of ICBs, targeting mainly the PD-L1/PD-1 axis, have been recently performed in early-stage and advanced TNBC disease [15,16,17,18,19,20,21,22,23,24]. This includes the phase 3 IMpassion130 trial [23], leading the Federal Drug Administration to approve in 2019 the anti-PD-L1 antibody atezolizumab combined with Nab-paclitaxel for the treatment of PD-L1-positive unresectable locally advanced or metastatic TNBC patients [25]. 

Overall, the different clinical trials that targeted the PD-L1/PD-1 axis demonstrated a relatively low-to-moderate improvement in objective response rates in TNBC patients; in most cases, effectiveness was increased by chemotherapies, possibly due to the exposure of neo-antigens, leading to increased activation of anti-tumor immune responses [11,14,15,16,17,18,19,20,21,22,23]. The relatively limited efficacy of ICBs in TNBC patients calls for identification of the mechanisms leading to poor impact of immunotherapies in this disease subtype. So far, it was assumed that the failure is due to immune-related mechanisms that prevent ICBs from reaching optimal effect, such as low T cell infiltration to tumors, the exhausted state of T cells, the activity of compensatory inhibitory mechanisms and the inability to optimally identify the patients who may benefit from ICB treatment.

In the current study, we investigated a different mechanism that may contribute to the limited efficacy of ICBs in TNBC patients. Our hypothesis in this study was that PD-L1, which is expressed by tumor cells in many TNBC patients [26,27,28,29,30], exerts cell-autonomous activities in the cancer cells, promoting their pro-metastatic potential. These activities are expected to remain intact when antibodies against PD-1 are used to block the immune suppressive activities of the PD-L1/PD-1 axis, because the pro-cancer functions of PD-L1 would remain untouched by the treatment and would continue fueling the pro-metastatic activities of the cancer cells. In this case, the therapeutic effects of ICBs directed to PD-1 could be potentiated by immunotherapies directed to PD-L1. In parallel, when antibodies against PD-L1 are used, it is possible that they are unable to efficiently block the two different arms of PD-L1-mediated activities simultaneously: PD-L1-induced immune suppression and pro-metastatic intrinsic activities in the cancer cells. In this case, ICBs targeting PD-L1 may reach maximal efficacy in inhibiting the cell-autonomous pro-metastatic effects of PD-L1 in the cancer cells if they will be given alongside ICBs that prevent immune suppression by targeting PD-1 or CTLA-4. Thus, in TNBC patients whose tumors express PD-L1, dual blocking strategies may improve efficacy, e.g., when ICBs directed to PD-L1 are joined by ICBs directed to PD-1 or CTLA-4. 

To date, only a limited number of publications have addressed the possibility that PD-L1 would act in an autonomous manner in breast cancer cells; these reports have used PD-L1 down-regulation or inhibition and demonstrated intrinsic PD-L1 activities in a few aspects of malignancy [31,32,33,34]. In our study, we extended these observations by taking the approach of up-regulating PD-L1 expression, in multiple breast tumor cell lines. This methodology enabled us to demonstrate not only that PD-L1 exhibits cell-autonomous activities that promote pro-metastatic functions of breast tumor cells in culture and in vivo, but also that the intensity of cell-intrinsic activities of PD-L1 is increased when PD-L1 levels are elevated. By using the experimental approach of WT-PD-L1 over-expression, we also held the advantage of analyzing PD-1 effects; these analyses have shown that PD-1 has promoted all pro-metastatic activities in WT-PD-L1 cancer cells. Moreover, we have provided evidence of PD-L1-induced chemokine-mediated autocrine cascades that promote breast tumor cell invasion and have also identified, for the first time, the S283 residue of PD-L1 as an indispensable regulator of these PD-L1 activities, in culture and in vivo. 

Together, our observations shed light on novel roles of PD-L1 that increase its impact as a major promoter of tumor progression in breast cancer. We revealed that, in parallel to the well-described activities of PD-L1 as an inhibitor of anti-tumor T cell activities, it also up-regulates intrinsic pro-metastatic cancer cell functions that are further promoted by PD-1. Our study holds major clinical implications, particularly in TNBC, as they propose that the joint activities of the immune suppressive and cell-intrinsic, pro-metastatic, activities of PD-L1 may explain the low response rate of many TNBC patient tumors to ICB treatments. In such a setting, patients whose cancer cells express PD-L1 could benefit from therapy approaches that target the immune suppressive activities of immune checkpoints such as PD-1 and CTLA-4, alongside with inhibition of the cell-autonomous functions of PD-L1, which would allow for a stronger therapeutic impact in TNBC.

## 2. Results

### 2.1. PD-L1 Exerts Cell-Autonomous Metastasis-Supporting Activities That Are Increased by Exposure of Breast Tumor Cells to PD-1

**Figure 1:**
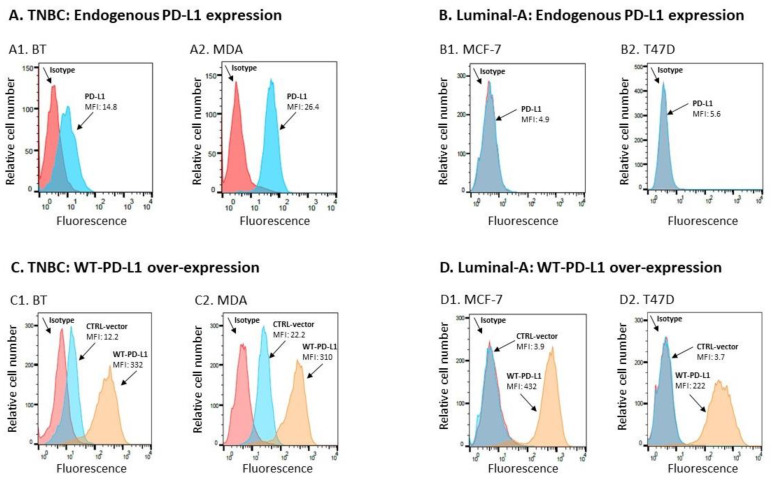
**PD-L1 expression levels, at endogenous levels and after WT-PD-L1 over-expression.** (**A**,**B**) Endogenous expression of PD-L1 by breast cancer cells. (**A**) TNBC cells: (**A1**) BT-549 (BT) cells. (**A2**) MDA-MB-231 (MDA) cells. (**B**) Luminal-A cells: (**B1**) MCF-7 cells. (**B2**) T47D cells. (**C**,**D**) PD-L1 expression by breast tumor cells following infection with PD-L1 or its control vector (CTRL-vector cells). (**C**) TNBC cells: (**C1**) BT cells. (**C2**) MDA cells. (**D**) Luminal-A cells: (**D1**) MCF-7 cells. (**D2**) T47D cells. In all parts, PD-L1 expression was determined by flow cytometry. Non-relevant isotype-matched antibodies served as negative controls (Isotype). MFI, mean fluorescence intensity. In parts (**C**,**D**), three cell types are demonstrated for each cell line: (1) “CTRL-vector”—Cells infected to express the control vector of PD-L1, stained by antibodies to PD-L1; these cells demonstrate endogenous PD-L1 expression (BT and MDA cells) or the lack of PD-L1 endogenous expression (MCF-7 and T47D cells), as appropriate; (2) “WT-PD-L1”: Cells infected to over-express WT-PD-L1, stained by antibodies to PD-L1; (3) Isotype—Cells stained by a non-relevant isotype-matched antibody control (no staining was noted for both WT-PD-L1 and CTRL-vector cells stained by isotype control; for simplicity, only one of two histograms is shown). The Figure represents multiple experiments that were performed for each cell type.

In this research, we used two systems in order to explore the cell-autonomous and PD-1-induced activities of PD-L1 in breast cancer cells: 

**(1) System 1**: Human TNBC cells, namely BT-549 (BT) and MDA-MB-231 (MDA) cells. In line with published reports on the expression of PD-L1 by TNBC cell lines [24,27,28,34], these two TNBC cell types express endogenous PD-L1 in a constitutive manner (Figure 1A). Here, we generated BT and MDA cells that over-expressed wild type (WT)-PD-L1, termed herein WT-PD-L1-BT and WT-PD-L1-MDA cells (Figure 1C). Because the sham vector-infected cells, called herein CTRL-vector cells, express endogenous PD-L1 but at lower levels than the WT-PD-L1-expressing cells, this system enabled us to determine to what extent the autonomous activities of PD-L1 depend on its expression levels, and also to reveal how exposure to PD-1 affects PD-L1 activities. 

**(2) System 2:** Human luminal-A breast cancer cells, namely MCF-7 and T47D cells. Luminal-A breast tumors are less aggressive than TNBC tumors [1,2,3,4,5]; it has been previously demonstrated that the incidence of PD-L1 expression is lower in luminal-A patients than in TNBC patients, and that luminal-A cells in culture express very low levels, if any, of endogenous PD-L1 [26,27,28,29,30]. Indeed, in Figure 1B we demonstrate that the luminal-A cell lines MCF-7 and T47D cells do not express endogenous PD-L1. In these cells, we have over-expressed WT-PD-L1 (Figure 1D), generating WT-PD-L1-MCF-7 and WT-PD-L1-T47D cells. These cells enabled us to isolate the cell-autonomous effects of PD-L1 and its PD-1-induced activities in a “naive” system in which background signals, driven by endogenous PD-L1, are absent. 

Based on reports demonstrating that soluble PD-1 is connected to an advanced disease state in different malignancies and is functional in activating PD-L1 (as indicated for example by regulation of immune activities) [35,36,37,38,39], the effects of PD-1 in both systems were determined by exposing the cancer cells to a soluble PD-1 protein. 

To determine the roles of PD-L1 in regulating pro-metastatic functions in breast cancer cells, we first analyzed the aspect of tumor cell growth. To this end, WT-PD-L1-BT and WT-PD-L1-MDA TNBC cells were cultured in similar numbers to CTRL-vector cells for four or five days. Tumor cell growth was determined at these two time points by cell counting, and not by XTT/MTT assays, as the latter may provide evidence to increased metabolic activities and not necessarily to tumor growth, as we have shown in our previous study [40].

**Figure 2:**
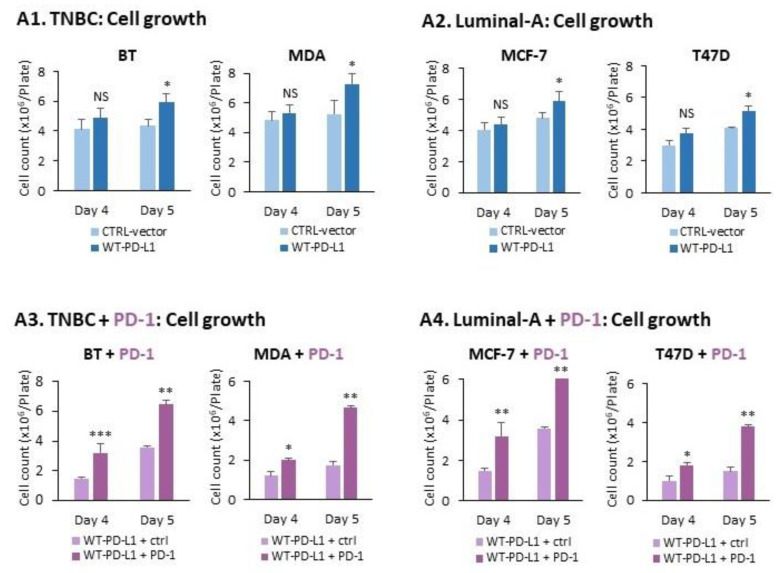

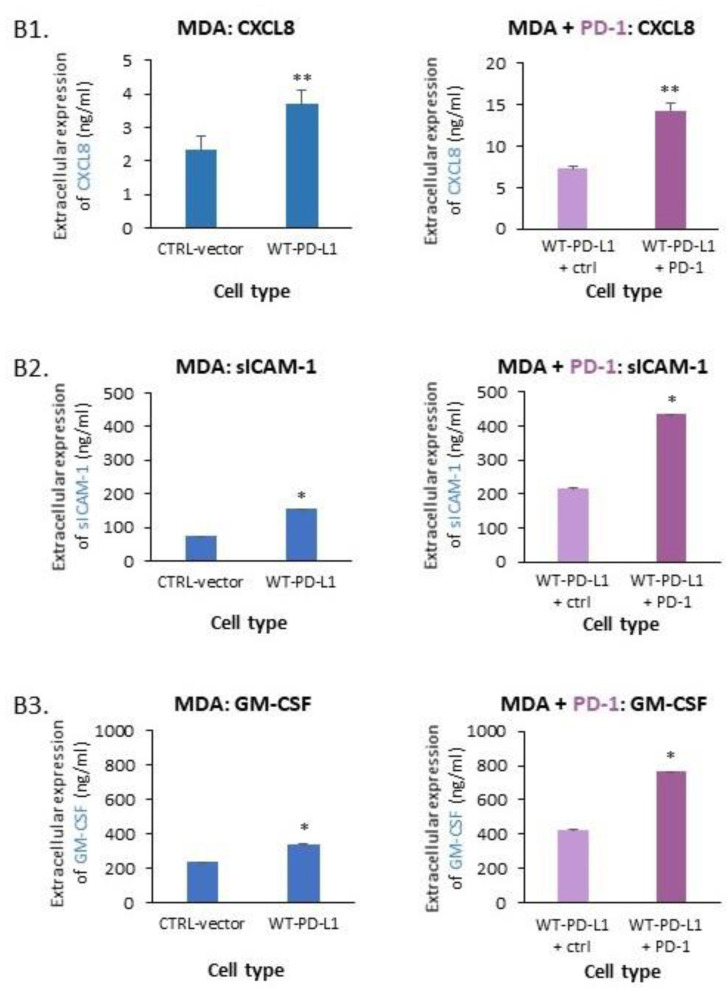
**The cell-autonomous activities of WT-PD-L1 increase pro-metastatic activities, and are potentiated by cell exposure to PD-1**. (**A1**,**A2**) Cell growth of WT-PD-L1-TNBC cells and WT-PD-L1-luminal-A cells and their corresponding CTRL-vector cells. (**A1**) TNBC cells: BT and MDA cells. (**A2**) Luminal-A cells: MCF-7 and T47D cells. WT-PD-L1-cells of each cell line were plated at similar concentration as the CTRL-vector cells. At days four and five after cell plating, the cells were counted in order to determine cell growth, as described in Section 4. (**A3**,**A4**) The impact of exposure to PD-1 on cell growth. (**A3**) TNBC cells: BT and MDA cells. (**A4**) Luminal-A cells: MCF-7 and T47D cells. PD-1 or its control (ctrl) (See Section 4 for more details) were added one day after cell plating, at 2 μg/mL for 72 h or 96 h (namely days four and five after cell plating). Cell growth was determined as described in Part (**A**) of the Figure. (**B**) The Figures in this part demonstrate the extracellular expression levels of pro-metastatic factors by WT-PD-L1-MDA cells and CTRL-vector-MDA cells. The cells were cultured, and 24 h later, PD-1 or its control (ctrl), were added as stated above. Cleared cell supernatants were collected and ELISA assays were performed to determine the expression of (**B1**) CXCL8; (**B2**) sICAM-1; (**B3**) GM-CSF. Each panel demonstrates the results of a representative experiment out of *n* = 3. *** *p* < 0.001, ** *p* < 0.01, * *p* < 0.05. NS, Non-significant.

At the fifth day of cell culture, the growth of WT-PD-L1-BT and WT-PD-L1-MDA cells was significantly higher than of their CTRL-vector counterpart cells (Figure 2(A1)). Similar findings were noted in luminal-A MCF-7 and T47D cells expressing WT-PD-L1, compared to their CTRL-vector cells (Figure 2(A2)). Together, these findings indicate that PD-L1 expression *per se* gives rise to a growth advantage of the cells (MCF-7 and T47D findings); moreover, the promotion of tumor cell growth was increased when the expression levels of PD-L1 were elevated (as demonstrated in BT and MDA cells). 

Then, tumor cell growth was determined following exposure of the cancer cells to PD-1 or its control, termed herein “ctrl”. Here, PD-1 and its ctrl were added to the cells one day after plating; cell growth was determined after an additional 72 or 96 h, namely days four and five after cell culturing. The findings of Figure 2(A3) demonstrate that exposure to PD-1 has effectively increased the growth of WT-PD-L1-BT and WT-PD-L1-MDA TNBC cells, at both time points. Similar findings were obtained with the luminal-A cells at both time points (Figure 2(A4)). Together, these findings indicate that PD-L1 provides not only autonomous but also PD-1-induced growth advantages to breast tumor cells. 

Studies measuring the release of pro-metastatic soluble factors by breast cancer cells were also performed, focusing on: (1) CXCL8 (IL-8): A potent chemoattractant of pro-metastatic neutrophils to tumor sites; this chemokine also induces metastasis-promoting functions in the cancer cells, such as invasion [41,42,43,44,45,46,47,48]; (2) sICAM-1 (soluble intercellular adhesion molecule 1): A pro-inflammatory mediator that was associated with increased progression in breast cancer patients [49,50,51]; (3) GM-CSF (granulocyte-macrophage colony stimulating factor): A pleotropic cytokine that elevates the expansion and recruitment of myeloid-derived suppressor cells that contribute to immune suppression in tumors [52,53]. Due to very low expression levels of these factors in BT and luminal-A cells, this part of the study was performed only with MDA cells. 

The findings of these analyses indicated that the increased expression levels of PD-L1 in MDA cells, compared to CTRL-vector-MDA cells have given rise to an elevated amount of all three factors: CXCL8, sICAM-1 and GM-CSF in the cell cultures (Figure 2(B1–3)). Moreover, these effects were promoted by PD-1 as indicated by the fact that the levels of CXCL8, GM-CSF and sICAM-1 were increased in WT-PD-L1-MDA cells following exposure to PD-1, compared to cells that were exposed to its ctrl (Figure 2(B1–3)). These findings demonstrate that the cell-autonomous activities of PD-L1 are increased with its elevated expression levels and are further potentiated by PD-1. 

**Figure 3:**
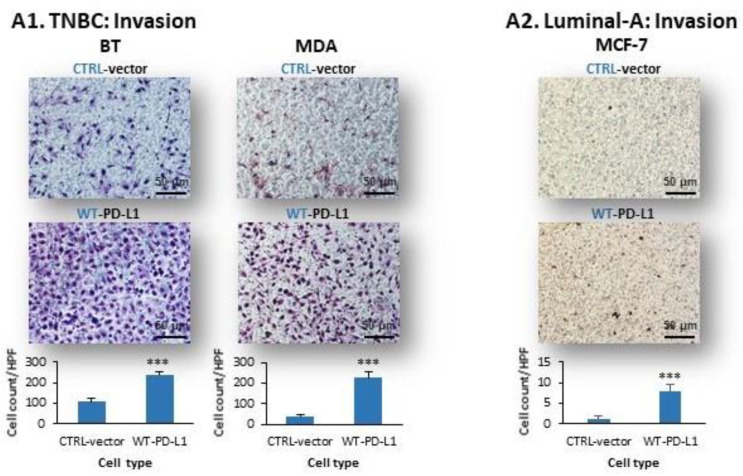

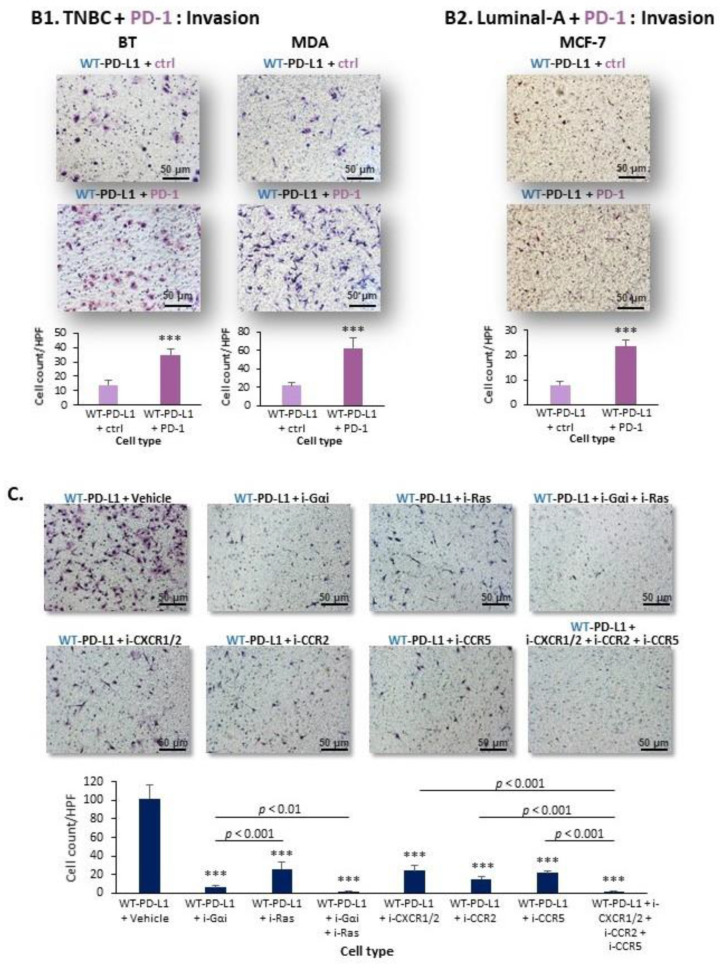
**The cell-autonomous activities of WT-PD-L1 increase breast tumor cell invasion, are potentiated by cell exposure to PD-1, and are led via molecular pathways that include Gαi- and Ras-mediated signaling and activation of chemokine receptors**. (**A**) Tumor cell invasion. (**A1**) Invasion of WT-PD-L1-TNBC cells: BT and MDA cells. (**A2**) Invasion of WT-PD-L1-luminal-A MCF-7 cells. Cell invasion was determined in matrigel-coated transwells, for the following time points: BT cells: 8 h; MDA cells: 11 h; MCF-7 cells: 21.5 h. (**B**) Tumor cell invasion following exposure to PD-1 or its control (ctrl). (**B1**) BT and MDA cells. (**B2**) MCF-7 cells. T47D cells were not analyzed due to lack of invasive abilities under the experimental conditions that were assayed. WT-PD-L1-over-expressing cells of each cell line were cultured, and 24 h later, PD-1 or its control (ctrl) were added at 2 μg/mL for additional 72 h. Then, cell invasion was determined in matrigel-coated transwells, at the time points indicated above. Note: In BT and MDA experiments, the number of cells loaded in the transwells was lower than in Part (**A**), in order to enable determination of increment in invasion by the addition of PD-1 when membranes are not over-loaded with cells. (**C**) The Figure demonstrates the effects of different pathway/receptor antagonists on invasion of WT-PD-L1-MDA cells. The cells were grown with the following inhibitors for 48 h: (1) i-Gαi—PTx; (2) i-Ras—FTS (Salirasib); (3) i-CXCR1/2—Reparixin; (4) i-CCR2—CAS 445479-97-0; (5) i-CCR5—Maraviroc. DMSO was used as a vehicle control (Vehicle). Cell invasion was determined in matrigel-coated transwells. Inhibitor concentrations (provided in Section 4) that did not affect cancer cell viability were selected based on titration analyses (see a comment on i-CCR2 in Section 4). HPF, High power field. Images (Bar, 50 μm) and graph demonstrate the results of a representative experiment out of *n* = 3. *** *p* < 0.001 for comparisons between the different treatments and the vehicle control.

Another pro-metastatic function that we have studied included the invasion of breast tumor cells through matrigel towards serum proteins. Here, a large increment in the number of invading cells in WT-PD-L1-BT and WT-PD-L1-MDA TNBC cells was noticed compared to their CTRL-vector counterparts that expressed endogenous PD-L1 levels (Figure 3(A1)). These findings indicate that the intrinsic invasion-promoting activities of PD-L1 can reach increased potency when the expression levels of the protein by the cells are elevated.

In line with previous reports demonstrating lower migratory properties of MCF-7 and T47D luminal-A cells compared to TNBC cells (such as MDA cells) [54,55], the invasion potency of luminal-A MCF-7 cells was weaker than of BT and MDA cells (Figure 3(A2) vs. Figure 3(A1)); however, even under these conditions of relatively lower migratory capacities, the invasion of MCF-7 cells was increased by PD-L1 over-expression (Figure 3(A2)) (T47D cells were not analyzed because they did not migrate at all in culture). These findings clearly illustrate the advantage given to the tumor cells, in terms of invasion, by the expression of PD-L1.

Moreover, exposure of WT-PD-L1-BT and WT-PD-L1-MDA TNBC cells to PD-1 (Figure 3(B1)), as well as of WT-PD-L1-MCF-7 luminal-A cells (Figure 3(B2)) increased the number of invading cells when they were compared to cells exposed to ctrl; please note that in these experiments of BT and MDA cells (Figure 3(B1)), the cells were plated in the transwells in lower numbers than in the experiments of Figure 3(A1), in order to prevent membrane overloading by tumor cells in the case that PD-1 would increase tumor cell migration. Thus, the above findings reveal that WT-PD-L1 effectively promoted tumor cell invasion in an autonomous manner, and more so upon exposure to PD-1.

We extended these experiments by determining the invasion capacity of the cancer cells in the context of extracellular matrices (ECM) that are typical of breast cancer metastatic sites. Because lungs are the most prevalent metastatic site in TNBC [56], the invasion of BT cells through human lung ECM was determined (Figure S1A). In parallel, in view of bones being a favorable metastatic site in luminal-A tumors [56], invasion of luminal-A MCF-7 cells through human bone ECM was analyzed (Figure S1B). In both cell types, WT-PD-L1 demonstrated autonomous activities that led to increased tumor cell invasion, which were further increased by exposure to PD-1 (Figure S1). 

Overall, the findings presented in this part of the study indicate that the expression of PD-L1, per se, elevated the pro-metastatic activities of breast tumor cells: cell growth, the expression of pro-metastatic mediators and invasion through matrigel and organ-relevant ECM. Moreover, these autonomous activities of WT-PD-L1 were increased when PD-L1 expression levels were elevated in the cells and were potentiated by exposure to PD-1. Thus, the cell-autonomous and PD-1-induced functions of PD-L1 can lead to exacerbated levels of pro-metastatic activities that are expressed by the tumor cells (proliferation and invasion) and that could affect the tumor microenvironment (CXCL8, sICAM-1, GM-CSF).

### 2.2. Cell-Autonomous PD-L1-Induced Invasion of TNBC Cells Is Promoted by Chemokine Axes

Next, to identify the molecular mechanisms that are involved in the cell-autonomous activities of PD-L1 in breast tumor cells, we inhibited in WT-PD-L1-MDA cells two major molecular components that hold high importance in cancer progression. The first component was the heterotrimeric G protein Gαi that mediates the signals delivered by a large variety of G protein-coupled receptors (GPCRs) [57,58,59]; in this case, the inhibitor was pertussis toxin (PTx), termed herein i-Gαi. The second signaling component was the Ras protein, which is stimulated typically by receptor tyrosine kinases (RTKs) [60,61,62]; Ras was blocked by FTS (Salirasib), which is termed herein i-Ras. i-Gαi and i-Ras were used in concentrations that did not cause tumor cell death, based on titration analyses (more details are provided in Section 4). 

Transwell studies determining the invasion of WT-PD-L1-MDA cells through matrigel demonstrated that i-Gαi and i-Ras, each alone, have given rise to 90–95% and 60–70% inhibition of tumor cell invasion, respectively (depending on the experiment) (Figure 3C). When the two inhibitors were combined, the invasion of TNBC cells was completely blocked (Figure 3C), indicating that the cell-autonomous activities of PD-L1 are mediated via Gαi- and RTK-driven signals, leading to elevated TNBC cell invasion, and that the processes are interconnected in regulating the PD-L1-enhanced invasion of the cells. 

**Figure 4:**
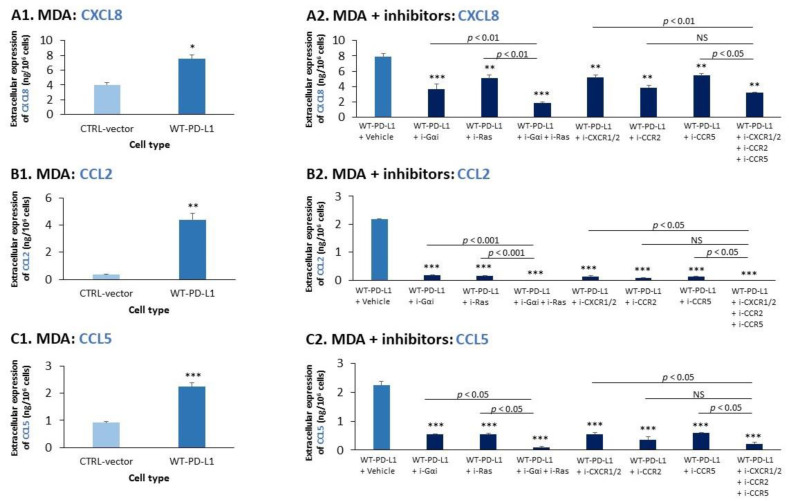
**The cell-autonomous activities of WT-PD-L1 act via chemokine receptors to upregulate the expression of their corresponding chemokines**. (**A1**,**B1**,**C1**) The extracellular expression of pro-metastatic chemokines by WT-PD-L1-MDA cells compared to their CTRL-vector-MDA counterparts, determined by ELISA. (**A1**) CXCL8 (the experiment shown here is different from the one presented in Figure 2(B1); the results are presented again for readers’ convenience). (**B1**) CCL2. (**C1**) CCL5. (**A2**,**B2**,**C2**) The effects of different pathway/receptor inhibitors on the expression of pro-metastatic chemokines by WT-PD-L1-MDA cells. DMSO was used as a vehicle control (Vehicle). The extracellular expression of CXCL8 (**A2**), CCL2 (**B2**) and CCL5 (**C2**) was determined in cleared supernatants by ELISA. Selection of Inhibitor concentrations and doses used are the same as in Figure 3C. The data demonstrate the extracellular levels of the chemokines, normalized to cell numbers. Each panel demonstrates the results of a representative experiment out of *n* = 3. *** *p* < 0.001, ** *p* < 0.01, * *p* < 0.05 and NS, Non-significant for comparisons between the different treatments and the vehicle control.

The prominent roles of Gαi-induced signaling in mediating the invasion of PD-L1-expressing TNBC cells has led us to determine which GPCRs may be involved in this process. So far, in the course of our studies we have demonstrated that WT-PD-L1 caused elevated CXCL8 levels in MDA cells (Figure 2(B1)). In line with it being a chemokine that promotes migratory processes, CXCL8 was found in the past to elevate tumor cell invasion, often through autocrine circuits [41,42,43,44,45,46,47,48]. CXCL8 activities are mediated by CXCR1 and CXCR2, which signal via Gαi [63], making this chemokine and its receptors possible candidates that are involved in the invasion of WT-PD-L1-MDA cells. Similarly, we hypothesized that the chemokines CCL2 and CCL5—which are pro-metastatic chemokines that promote tumor cell invasion [41,42,46,48,64,65,66,67,68,69,70,71] and act mainly through the Gαi-signaling receptors CCR2 and CCR5, respectively [63,72,73]—also contribute to increased invasion of WT-PD-L1-MDA cells. Supporting this possibility are reports demonstrating that CXCR1/2, CCR2 and CCR5 are expressed by MDA cells [74,75,76,77,78] and by our findings, demonstrating that WT-PD-L1-MDA cells expressed higher levels of CCL2 and CCL5 than their respective CTRL-vector cells (Figure 4(A1,B1,C1)); for the readers’ convenience, the figure demonstrates another example of this effect for CXCL8 expression). 

Based on this information, we asked if CXCR1/2, CCR2 and CCR5 participate in the signaling process through which WT-PD-L1 leads to increased invasion of MDA cells. Therefore, we downregulated the activities of these receptors by antagonists that are conventionally used to block their activities, in research and also in the clinic (e.g., the CCR5 antagonist); the inhibitors were used in concentrations that were selected by titration analyses, guaranteeing no effects on cancer cell viability (or partial effect in the case of CCR2 inhibitor, as described in Section 4). The following inhibitors were used: (1) Reparixin—called herein i-CXCR1/2—was used to inhibit signaling mediated by CXCR1/2, the receptors of CXCL8; (2) CAS 445479-97-0, which is termed herein i-CCR2, inhibits CCR2, the main receptor that mediates CCL2 signaling; (3) Maraviroc, called herein i-CCR5, was employed to antagonize the functions of CCR5, a key receptor mediating CCL5-induced signals. 

The results of Figure 3C demonstrate that each of the studied receptors—CXCR1/2, CCR2 and CCR5—induced intracellular signals that were necessary for WT-PD-L1-induced invasion. WT-PD-L1-MDA cells that were treated by i-CXCR1/2 demonstrated reduction of 75 ± 3.6% in invasion. In parallel, i-CCR2 or i-CCR5 caused 85 ± 2.6% and 75 ± 2.4% inhibition of WT-PD-L1-MDA cell invasion, respectively. The inability of each receptor to compensate for the absence of the signals delivered by the other receptors suggests that all receptors, CXCR1/2 + CCR2 + CCR5, needed to be activated simultaneously in order to mediate the intrinsic signals of WT-PD-L1 that lead to increased cancer cell invasion. Accordingly, when all three inhibitors were used together, invasion of WT-PD-L1-MDA cells was completely blocked (Figure 3C).

The above findings have motivated us to explore the possibility that the signaling by CXCR1/2, CCR2 and CCR5 is inhibited, at least partly, because of reduced availability of their corresponding chemokine ligands. If indeed so, we would expect to see reduced extracellular levels of CXCL8, CCL2 and/or CCL5 produced by WT-PD-L1-MDA cells in the presence of the inhibitors.

To determine this possibility, we first asked if the cell-autonomous activities of WT-PD-L1—which led to elevated extracellular levels of CXCL8, CCL2 and CCL5 in WT-PD-L1-MDA cells compared to CTRL-vector cells (Figure 4(A1,B1,C1))—depended on the large families of receptors signaling via Gαi and Ras (results were normalized to cell numbers). As shown in Figure 4(A2,B2,C2), i-Gαi caused 50–55%, 90–95% and 75–85% inhibition of CXCL8, CCL2 and CCL5 expression, respectively. In parallel, treatment of the tumor cells by i-Ras also led to a reduction of their extracellular levels: 35–40%, 90–95% and 75–85% inhibition of CXCL8, CCL2 and CCL5, respectively. Of note, complete inhibition of CCL2 and CCL5 production (90–100% reduction) and potent inhibition of CXCL8 levels (75–80%) were obtained when both inhibitors—i-Gαi and i-Ras—were used together (Figure 4(A2,B2,C2)).

Further analyses indicated that signaling by the chemokine receptors CXCR1/2, CCR2 and CCR5 regulated the expression of their own corresponding ligands, CXCL8, CCL2 and CCL5 (results were normalized to cell numbers). The findings of Figure 4(A2) demonstrate that the extracellular levels of CXCL8 were reduced by 25–35% when WT-PD-L1-MDA cells were treated by i-CXCR1/2, by 55–65% when i-CCR2 was used and by 35–45% when i-CCR5 was employed. The levels of CCL2 were even more effectively reduced by the three inhibitors: 85–90%, 90–95% and 85–90% inhibition by i-CXCR1/2, i-CCR2 and i-CCR5, respectively (Figure 4(B2)). Prominent reduction in CCL5 production was also noted upon treatment of WT-PD-L1-MDA cells by each of the three inhibitors: 70–80% by i-CXCR1/2, 75–85% by i-CCR2 and 70–80% inhibition by i-CCR5 (Figure 4(C2)).

Moreover, when all three inhibitors of the chemokine receptors were used together in WT-PD-L1-MDA cells, complete inhibition of CCL2 and almost full reduction of CCL5 extracellular expression were noted (Figure 4(B2,C2)). The production of CXCL8 was only partially blocked by the combined use of i-CXCR1/2, i-CCR2 and i-CCR5 together (60–65% inhibition; Figure 4(A2)), demonstrating a different regulation of CXCL8 compared to CCL2 and CCL5. 

These findings, demonstrating the roles of chemokine axes in regulation of the autonomous pro-metastatic activities of PD-L1 in TNBC, are supported by additional analyses that we have performed with the TCGA dataset of basal breast cancer patients (*n* = 141). Here, we found that the mRNA levels of PD-L1 were significantly associated with high mRNA expression levels of members of the CCL5-CCR5 and CCL2-CCR2 axes (Figure S2); in line with the experiments we have performed with Reparixin, shown in Figure 4, the correlation of PD-L1 with members of the CXCL8-CXCR1/2 axis was lower (Figure S2).

Together, these studies point at a mechanism that is induced by WT-PD-L1, in which it leads to an up-regulation of chemokine expression by the cancer cells. These chemokines then activate their corresponding receptors, leading to increased invasion of WT-PD-L1-MDA cells. Thus, positive-feedback autocrine loops that are mediated by the chemokine receptors CXCR1/2, CCR2, CCR5 and their ligands play key roles in promoting the invasion of MDA cells when WT-PD-L1 is expressed by the cells. 

### 2.3. The Cell-Autonomous and PD-1-Induced Activities of PD-L1 in Breast Tumor Cells Depend on Integrity of the S283 Residue

The sequence of PD-L1 lacks conventional signaling motifs and the domains that mediate its intracellular signals are far from being fully resolved. To date, three conserved intracellular domains were identified in PD-L1, two of which are connected to regulation of interferon-induced apoptotic responses [79,80]. Using the Phosphonet dataset (http://www.phosphonet.ca (accessed on 12 December 2021)), we observed that several PD-L1 residues may undergo phosphorylation; screening studies of cancer phospho-proteome by mass spectrometry indicated that the intracellular serine 283 (S283) residue is a key phosphorylation site in PD-L1 (https://www.phosphosite.org/proteinAction.action?id=19198&showAllSites=true (accessed on 12 December 2021)). The S283 residue is located at the region of the three conserved domains that were identified in PD-L1, underlined herein: RMMDVKKCGIQDTNSKKQ**S^283^**DTHLEET. Based on this information, we asked if S283 regulates the autonomous pro-metastatic functions of PD-L1 in breast cancer cells.

**Figure 5:**
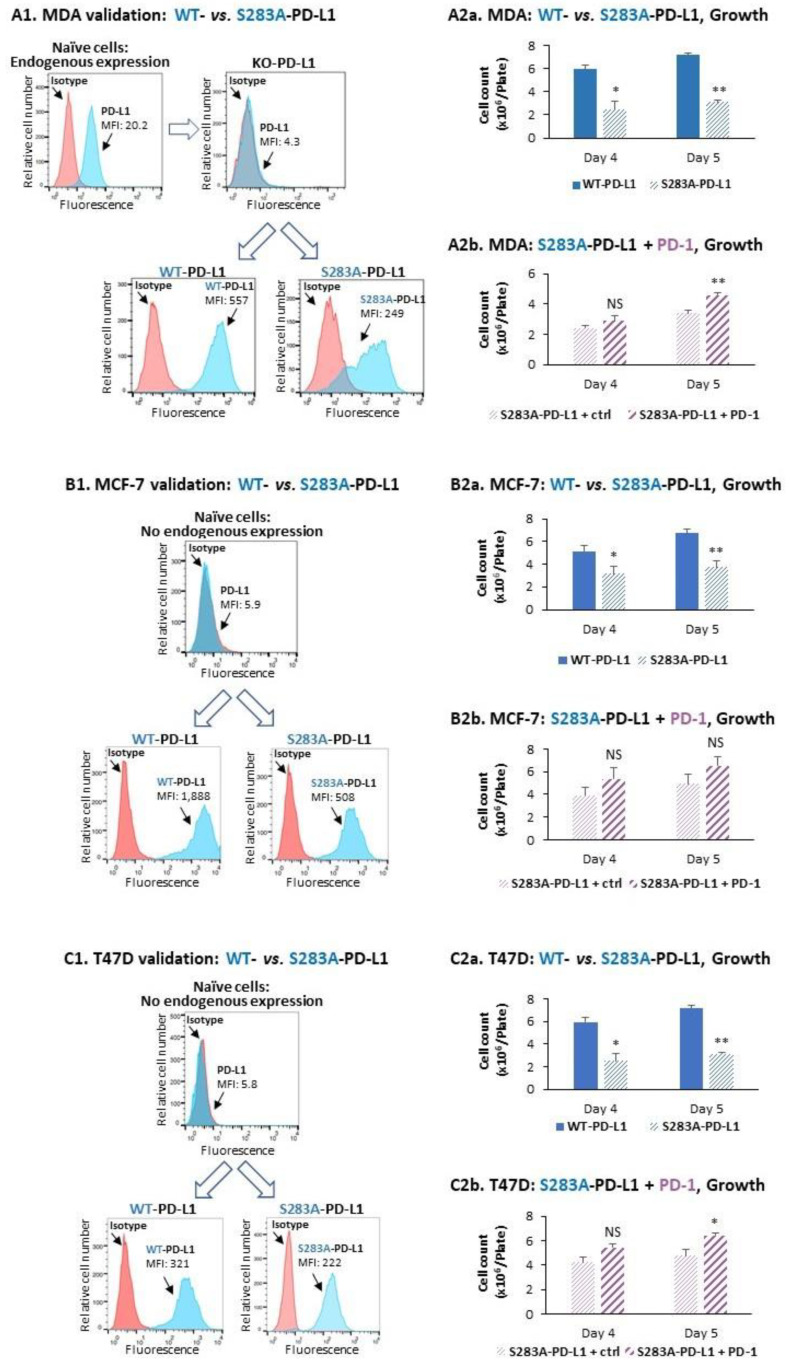
**The S283 residue of PD-L1 is required for optimal cell-autonomous and PD-1-induced tumor cell growth.** (**A**) Analyses of MDA cells. (**A1**) MDA cells expressing WT-PD-L1 or S283A-PD-L1. As MDA cells endogenously express PD-L1 (Figure 1(A2)), PD-L1 in these cells was knocked-out by the Alt-R CRISPR-Cas9 method and validated for the lack of PD-L1 expression by flow cytometry, as described in Figure S5. Then, these KO-PD-L1 cells were analyzed to guarantee an out-of-frame sequence of PD-L1 and were infected to express WT-PD-L1 or S283A-PD-L1; this step was followed by validation of PD-L1 cell surface expression in these two cell types by flow cytometry analyses, as shown in Figure 1. Control MDA cells in which PD-L1 was knocked out were infected by a sham vector; they did not express PD-L1 as shown in the Figure (called herein KO-PD-L1-MDA cells). (**A2**) Determination of MDA cell growth. (**A2a**) WT-PD-L1-MDA cells and S283A-PD-L1-MDA cells were plated at similar concentrations. At days four and five after cell plating, the cells were counted in order to determine cell growth. (**A2b**) One day after culturing of S283A-PD-L1-MDA cells, PD-1 or its ctrl were added at 2 μg/mL for 72 h or 96 h (namely, days four and five after cell plating); then, the cells were counted in order to determine cell growth. (**B**,**C**) Analyses of MCF-7 and T47D cells, respectively. (**B1**,**C1**) MCF-7 and T47D cells, which do not express PD-L1 constitutively (Figure 1(B1,2), respectively), were infected to express WT-PD-L1 (as shown in Figure 1D) or S283A-PD-L1 and were then analyzed by flow cytometry for the expression of PD-L1 at the cell surface. (**B2**,**C2**) Determination of cell growth. (**B2a**,**C2a**) WT-PD-L1-MCF-7 and WT-PD-L1-T47D cells vs. S283A-PD-L1-MCF-7 cells and S283A-PD-L1-T47D cells, respectively. (**B2b**,**C2b**) The effects of PD-1 on cell growth of S283A-PD-L1-MCF-7 cells and S283A-PD-L1-T47D cells. The experimental design was similar to that of Part Figure 5(A2). Panels (**A1**,**B1**,**C1**): Non-relevant isotype-matched antibodies served as negative controls (Isotype). MFI, mean fluorescence intensity. Panels (**A2**,**B2**,**C2**) demonstrate the results of a representative experiment out of *n* = 3. ** *p* < 0.01, * *p* < 0.05. NS, Non-significant.

To determine the roles of S283 in mediating PD-L1 pro-metastatic functions, we generated a mutated human PD-L1 variant in which S283 was replaced by alanine, and generated S283A-PD-L1-expressing cells that were tested in parallel to WT-PD-L1-expressing cells in functional pro-metastatic assays. First, in MDA cells that endogenously express PD-L1 (Figure 1(A2)), the Alt-R CRISPR-Cas9 system was used to knock-out (KO) the intrinsic expression of PD-L1 (Figure S5). The MDA cells, in which PD-L1 was knocked-out and did not express PD-L1 (data not shown), were then infected by WT-PD-L1 or S283A-PD-L1 (Figure 5(A1)) or by the corresponding sham vector (Figure S5). The cells that were infected by the sham vector were used as control and are called herein KO-PD-L1 cells (in order to distinguish them clearly from CTRL-vector cells used in the previous parts of the study) and did not express cell surface PD-L1 (Figure 5(A1)). 

In this setting of MDA cells, determination of cancer cell growth at days four and five after cell plating demonstrated 58 ± 6% and 57 ± 2% reduction in growth rate of S283A-PD-L1-MDA cells compared to WT-PD-L1-MDA cells, respectively (Figure 5(A2a)). Moreover, exposure of S283A-PD-L1-MDA cells to PD-1 elevated tumor cell growth (Figure 5(A2b)), but only at 1.2 ± 0.7-fold induction, compared to 1.9 ± 0.5-fold induction in WT-PD-L1 cells (Figure 2(A3)), at day five after cell plating. 

Then, S283A-PD-L1 was expressed in the luminal-A-based systems of MCF-7 and T47D cells (Figure 5(B1,C1), respectively); the growth of these cells was compared to WT-PD-L1-expressing cells that were already generated (Figure 1D). As demonstrated in Figure 5(B2a,C2a), the growth of S283A-PD-L1-MCF-7 cells and of S283A-PD-L1-T47D cells was significantly lower than the growth rate of their WT-PD-L1-MCF-7 and WT-PD-L1-T47D counterparts. Moreover, unlike its ability to promote the growth of WT-PD-L1-MCF-7 cells and of WT-PD-L1-T47D cells (Figure 2(A4)), PD-1 stimulation did not significantly increase the growth of S283A-PD-L1-MCF-7 cells (Figure 5(B2b)) and only minimally elevated the growth of S283A-PD-L1-T47D cells (Figure 5(C2b)).

**Figure 6:**
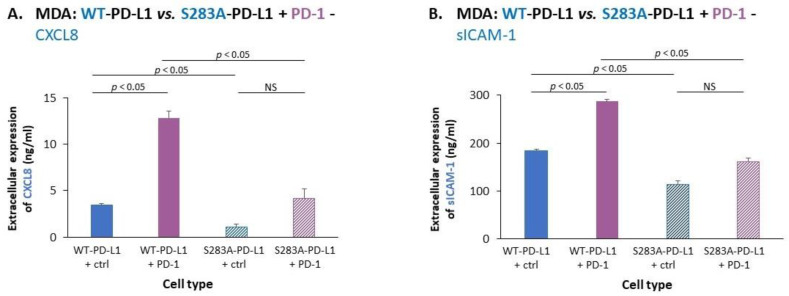
**The cell-autonomous and PD-1-induced expression of CXCL8 and sICAM-1 depend on the integrity of the S283 residue of PD-L1**. The extracellular expression levels of (**A**) CXCL8 and (**B**) sICAM-1 were determined in cell supernatants of WT-PD-L1-MDA and S283A-PD-L1-MDA cells by ELISA assays. PD-1 or its control (ctrl) were added at 2 μg/mL for 72 h. Each panel demonstrates the results of representative experiment out of *n* = 3. *p* values are demonstrated in the Figure. NS, Non-significant.

The expression of CXCL8 and sICAM-1 was also analyzed in this setting, with and without exposure to PD-1. The findings of Figure 6 demonstrate that the amounts of CXCL8 and sICAM-1 expressed by S283A-PD-L1-MDA cells were significantly lower than the protein levels expressed by WT-PD-L1-MDA cells; moreover, while elevating the levels of CXCL8 and sICAM-1 by WT-PD-L1-MDA cells, PD-1 did not significantly promote the amounts of these two mediators by S283A-PD-L1-MDA cells (Figure 6). 

**Figure 7:**
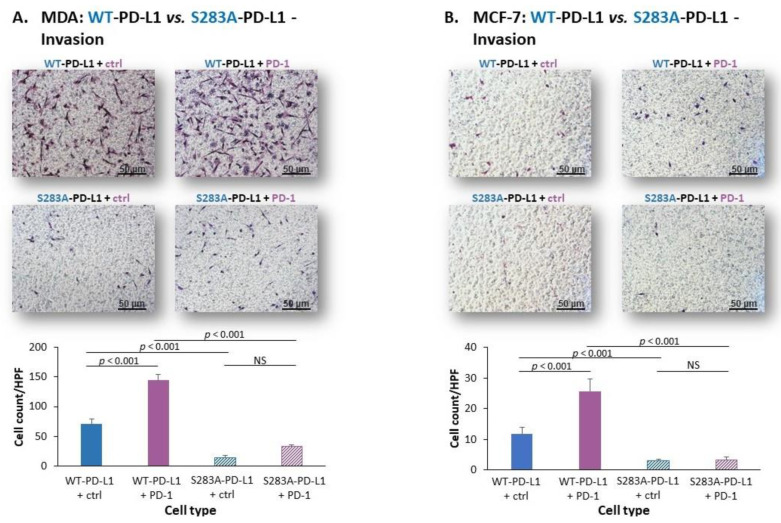
**The cell-autonomous and PD-1-induced activities of PD-L1 that promote tumor cell invasion depend on the integrity of the S283 residue of PD-L1.** Invasion of (**A**) MDA and (**B**) MCF-7 cells that over-expressed WT-PD-L1 or S283A-PD-L1 was determined in transwell assays, after exposure to PD-1 or its control (ctrl). PD-1 or its ctrl were added one day after cell plating, at 2 μg/mL, for 72 h. Cell invasion was determined in matrigel-coated transwells for the time points indicated in Figure 3A. HPF, High power field. Images (Bar, 50 μm) and graphs, in both panels (**A**,**B**) demonstrate the results of a representative experiment out of *n* = 3. *p* values are demonstrated in the Figure. NS, Non-significant.

Then, experiments analyzing invasion through matrigel demonstrated that the invasion of S283A-PD-L1-MDA cells and of S283A-PD-L1-MCF-7 cells was much lower than of their counterpart cells that expressed WT-PD-L1 (Figure 7A,B). Moreover, exposure to PD-1 led to a large increment in the number of invading WT-PD-L1-MDA cells and WT-PD-L1-MCF-7 cells, whereas only a small or no increase in invading cells was noted in PD-1-exposed S283A-PD-L1-MDA and S283A-PD-L1-MCF-7 cells (Figure 7A,B, respectively). Similar results were obtained for both MDA and MCF-7 cells when invasion through lung ECM or bone ECM (for MDA and MCF-7 cells, respectively) was studied (Figure S3A,B, respectively).

A recent study demonstrated that AMP-activated protein kinase (AMPK) phosphorylates the S283 residue of PD-L1 [81]. To follow up on this observation, we modeled the interactions of a 7-aa peptide of the carboxyl terminus domain of WT-PD-L1 (containing S283) and S283A-PD-L1 (containing A283) with the catalytic domain of AMPK. Figure S4A demonstrates the structure of AMPK (PDB ID 4RER); Figure S4(B1,2) demonstrate the predicted binding of AMPK catalytic domain to two peptides, one containing the S283 residue of WT-PD-L1 and the second containing the A283 residue of S283A-PD-L1, respectively. These analyses revealed a smaller distance between the catalytic aspartate of AMPK and S283 (that is found in WT-PD-L1) than with A283 (of S283A-PD-L1). Furthermore, MMGBSA calculations that were executed by Prime suggested that the binding efficiency of WT-PD-L1 to MAPK is better than of S283A-PD-L1 (Table S1).

Together, the observations presented in this part of the study provided the first evidence to the indispensable roles of S283 in mediating PD-L1 signaling and induction of pro-metastatic effects in breast cancer cells; they also illustrated the increased binding efficiency of WT-PD-L1 to AMPK, over the binding efficiency of mutated PD-L1, that carries a serine to alanine alteration.

### 2.4. The Cell-Autonomous Activities of PD-L1 Promote Tumor Progression In Vivo, in a S283-Dependent Manner

**Figure 8:**
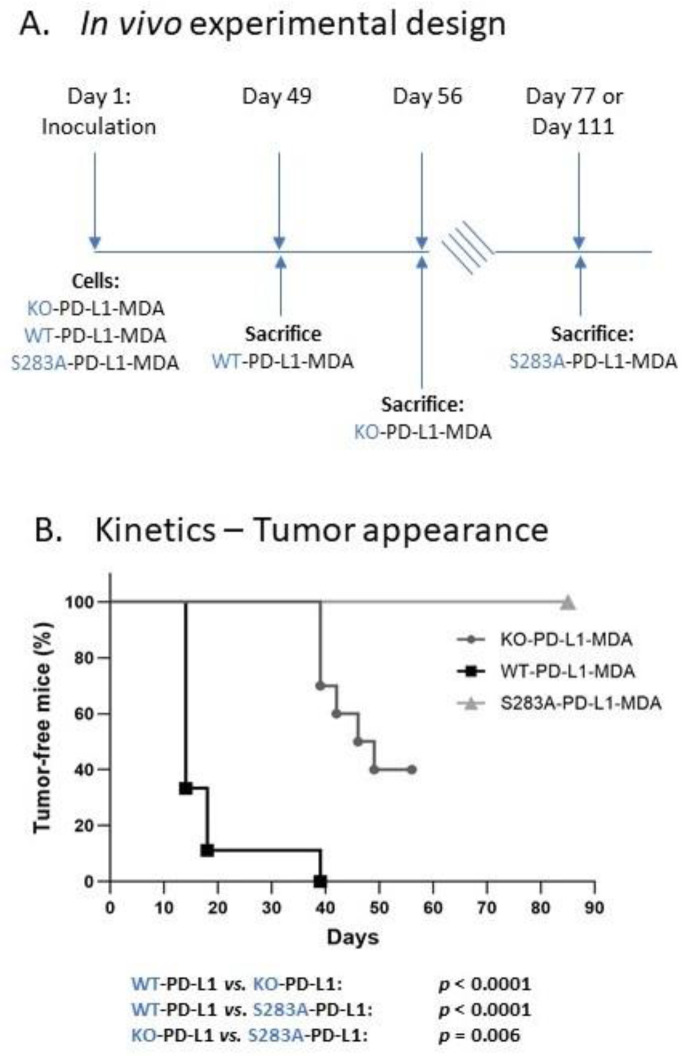
**The cell-autonomous activities of PD-L1 shorten the lag period of tumor appearance in vivo and depend on the integrity of the S283 residue of PD-L1.** (**A**) Description of the experimental procedure taken in order to determine the cell-autonomous roles of PD-L1 and its S283 residue in regulating tumor growth and metastasis. Tumor cells were administered to the mammary fat pad of female nude mice, deficient in T cell activities. This part of the Figure demonstrates the time points in which the mice of each group were sacrificed and analyzed for tumor volumes and metastasis formation. All cells were infected to express mCherry; tumor growth was determined by caliper every 3–4 days and was validated at different time points also by intravital imaging of the mammary region. Two independent experiments were performed, with the following mouse groups: (1) Group 1 = KO-PD-L1-MDA cells, with a total of *n* = 10 mice in the two experiments together. To follow up on the regulations of the Ethics Committee of Animal Use, the experiments of this group of mice were terminated at day 56 after tumor cell inoculation. Of note, as indicated in Figure 5(A1), in these cells the expression of endogenous PD-L1 was knocked out and the cells were infected with the control vector of PD-L1; thus, these cells do not express PD-L1 at all. (2) Group 2 = WT-PD-L1-MDA cells (generated as indicated in Figure 5(A1)), with a total of *n* = 9 mice in the two experiments together; the experiments of this group were terminated at day 49. (3) Group 3 = S283A-PD-L1-MDA cells (generated as indicated in Figure 5(A1)), with a total of *n* = 9 mice in the two experiments together, followed up until day 77. Follow up of five of the nine mice was continued until day 111. (**B**) Kinetics of tumor cell appearance. *p* values are demonstrated in the Figure.

To follow up on the above results, we asked if the cell-autonomous activities of PD-L1—that elevated pro-metastatic activities of TNBC cells—are potent enough to promote tumor growth and metastasis in vivo and, if so, if they depend on the S283 residue. To this end, we administered mCherry-expressing KO-PD-L1-MDA, WT-PD-L1-MDA and S283A-PD-L1-MDA cells to the mammary fat pad of female nude mice (Figure 8A). By using these mice, which are athymic and, thus, are deficient in T cell activities, we could ascertain that the impact of PD-L1 on tumor growth and metastasis, if observed, is due to its cell-autonomous activities and not because it induced T cell immune suppression. 

Here, we performed two independent experiments, including in total *n* = 9–10 mice in each of the following three groups: (Group 1) Mice injected with KO-PD-L1-MDA cells, which do not express PD-L1 at all (they were infected with control vector, as described in Figure 5(A1)); (Group 2) Mice injected with WT-PD-L1-MDA cells; (Group 3) Mice injected with S283A-PD-L1-MDA cells. 

**Figure 9:**
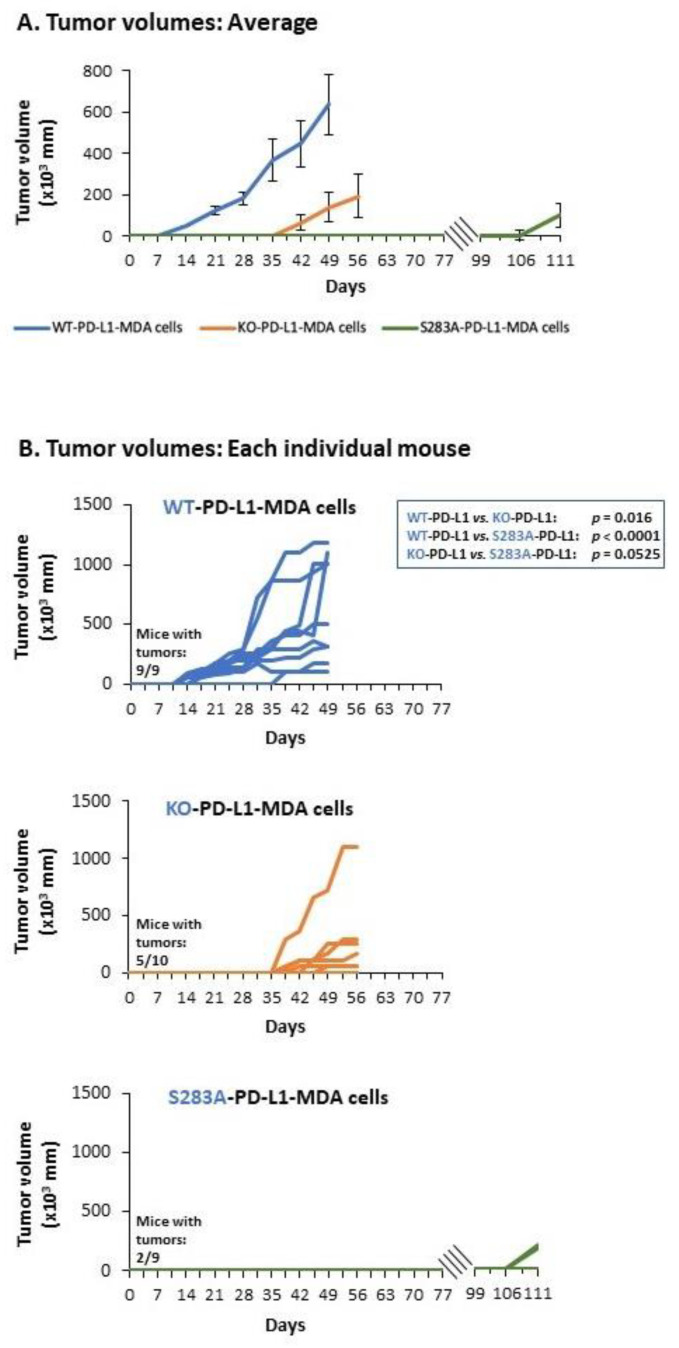
**The cell-autonomous activities of PD-L1 lead to increased tumor volumes in vivo and depend on the integrity of the S283 residue of PD-L1.** Tumor cells were administered to the mammary fat pad of female nude mice, as described in Figure 8A, including the same three groups of mice. The current Figure shows tumor volumes at different time points along the process, determined by caliper, measured every 3–4 days (validated at some of the time points by intravital imaging mCherry signals). (**A**) Averages of tumor volumes in each group of mice ± SEM, at each time point. (**B**) Each panel shows a different mice group, where tumor volume in each individual mouse is demonstrated along tumor progression. *p* values of comparisons between tumor volumes are indicated in the Figure.

The data of these experiments provide definite evidence to autonomous PD-L1 activities, which powerfully up-regulate tumor growth by TNBC cells. The WT-PD-L1-MDA cells (Group 2) exhibited a clear growth advantage over KO-PD-L1-MDA cells (Group 1), in terms of lag period until tumor appearance, as well as in tumor volume (Figure 8B and Figure 9). The first tumors appeared in the WT-PD-L1-MDA-injected mice 14 days after tumor cell inoculation; based on tumor sizes and regulations of the Ethics Committee for Animal Use, the mice of this group were sacrificed at day 49 (Figure 8A and Figure 9). In contrast, in KO-PD-L1-MDA-injected mice, tumors first appeared at day 39 after tumor cell inoculation and mice were sacrificed at day 56. Moreover, the S283 residue of PD-L1 was found to have a clear-cut role in mediating PD-L1 cell-autonomous pro-tumorigenic roles, as indicated by the fact that until day 77, none of the nine S283A-PD-L1-MDA-injected mice developed a palpable tumor (Group 3; Figure 8B and Figure 9). Here, we wish to indicate that in five of the nine mice, we could follow-up tumor growth until day 111; three of these mice did not develop primary tumors; in the other two, the tumors appeared at day 109 (Figure 8B and Figure 9). 

**Figure 10:**
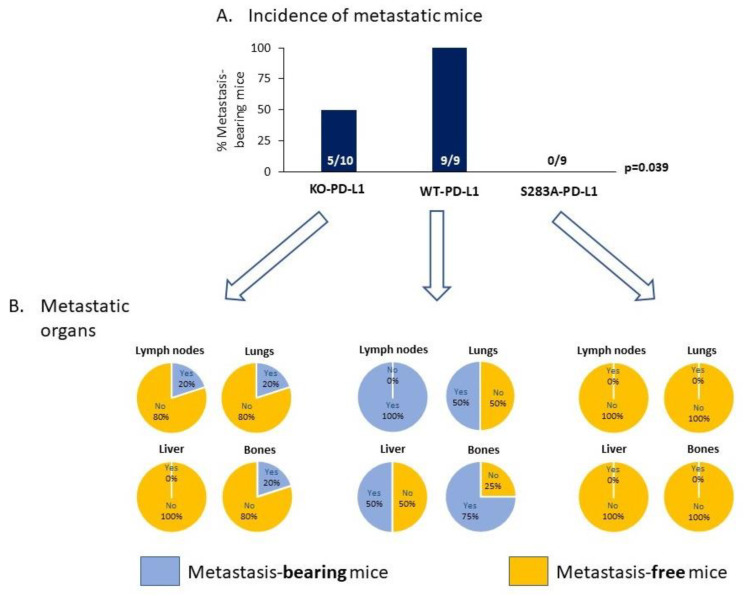
**The cell-autonomous activities of PD-L1 promote metastasis in vivo and depend on the integrity of the S283 residue of PD-L1.** (**A**) Incidence of metastasis-bearing mice. Metastases were determined in the three groups of mice at the time points that they were sacrificed: (1) Group 1 = KO-PD-L1-MDA cells: at day 56, in *n* = 10; (2) Group 2 = WT-PD-L1-MDA cells: at day 49, in *n* = 9; (3) Group 3 = S283A-PD-L1-MDA cells, at day 77 for four mice and at day 111 for five mice in which follow up was extended. Statistical analysis was performed for the three groups of mice at day 77 (in Group 3, analysis included 4 of the mice); of note, no metastases were detected in the five mice whose follow up was extended until day 111. *p* value is demonstrated in the Figure. (**B**) The incidence of mice bearing metastases in different organs, calculated in mice with metastases only.

Analyses of metastases in these three groups of mice were performed by intravital imaging and ex vivo analyses by using mCherry signals in the CRi Maestro device; these analyses were performed on the day in which the mice of each group were sacrificed. This investigation identified definitive roles for cell-autonomous PD-L1 activities in promoting metastasis formation, which were fully dependent on the S283 residue of the protein (Figure 10). Whereas 100% of the mice injected with WT-PD-L1-MDA cells developed metastases that were visible at day 49 (Group 2), only 50% of the KO-PD-L1-MDA-injected mice developed metastases, as determined at day 56 (Group 1). The roles of S283 in the PD-L1-mediated metastatic process were envisioned by the lack of metastases in all four mice injected with S283A-PD-L1-MDA cells (Group 3), which were followed until day 77; this observation was further enforced by the fact that in those five mice in which metastases were analyzed at day 111, no metastases were detected as well (Figure 10). 

The data of Figure 10 also indicate that WT-PD-L1-MDA cells (Group 2) lead to metastases in a broader range of organs than KO-PD-L1-MDA cells (Group 1). In 100% of WT-PD-L1-MDA-injected mice, metastatic foci were detected in lymph nodes located in proximity to the primary tumor; bone metastases were noted in 75% of the mice and, in 50% of the mice, metastases appeared in the lungs and liver. In contrast, in metastatic mice injected with KO-PD-L1-MDA cells, metastases appeared in low and equal incidence in tumor-adjacent lymph nodes, lungs and bones (20% in all organs); no metastatic foci were detected in the liver. 

Together, these findings indicate that the cell-autonomous tumor-supporting activities of PD-L1 promote tumor growth and metastases, and that the S283 residue is essential for its activities.

## 3. Discussion

The era of immunotherapy brings much promise to cancer treatment but also many challenges that require improved identification of the mode of action of inhibitory immune checkpoints in cancer. In the current study, we provide evidence to several novel findings on the roles of PD-L1 in breast cancer progression:

1. PD-L1 exerted tumor-promoting functions in breast cancer, which are not connected to its ability to induce immune suppression but rather to potentiation of intrinsic tumor cell activities that support tumor progression: cancer cell proliferation, release of soluble pro-metastatic factors and invasion through matrigel and organ-relevant ECM. Moreover, these cell-intrinsic pro-metastatic activities of PD-L1 were more potent when its levels were increased, indicating that not only the incidence of PD-L1-expressing cells but also its expression levels by the cells dictates the efficacy of PD-L1 in promoting metastasis-supporting intrinsic activities in the cancer cells. Furthermore, PD-1 enhanced all of these activities and the cell-autonomous activities of PD-L1 potently increased tumor growth and metastasis in the in vivo setting, independently of its immune suppressive activities.
2. We demonstrated, for the first time, that the S283 residue of PD-L1, which is expressed at the intracellular domain of the protein, is absolutely essential for PD-L1-induced intracellular signaling that leads to increased tumor cell proliferation, release of pro-metastatic soluble factors and invasion. The cardinal roles of S283 were reinforced by in vivo studies, demonstrating the critical roles of the S283 residue in regulating the intrinsic activities of PD-L1 that lead to increased tumor growth and metastasis.
3. We identified novel intracellular signaling cascades that mediate PD-L1 intrinsic activities in TNBC cells. We demonstrated that chemokine axes can establish positive autocrine feedback loops that connect intracellular signaling events with the increased ability of TNBC cells to invade. Here, our research identified the chemokine receptors CXCR1/2, CCR2, CCR5 and their ligands to stand in the center of the regulatory circuits that control PD-L1 pro-tumoral activities in TNBC cells.

Our findings in this study add much to published reports on advantages given to tumor cells by expressing PD-L1. The approach generally taken so far was to knock-out/knockdown PD-L1 expression in breast cancer cells or to inhibit it by antibodies; such studies have shown that down-regulation of PD-L1 has led to inhibition of tumor cell proliferation and migration, to reduction in EMT-related properties of the cells and to the expression of CSC-related properties [31,32,33,34]. Under conditions of PD-L1 knock-down, the ability of TNBC cells to form primary tumors was not modified, but the metastatic process was inhibited [33]. 

To complement and expand the findings obtained by the downregulation/inhibitory approach, we took a different research direction in our study, using PD-L1 over-expression in several types of breast cancer cells. Here, we determined not only the effect of tumor cell-expressed PD-L1 on cancer cell properties but also whether its activities depend on its expression levels and on exposure to its ligand, PD-1. To this end, we followed published reports demonstrating relatively low incidence of PD-L1 expression in luminal-A patients compared to TNBC patients, and low/no expression in luminal-A cell lines, while revealing endogenous expression of PD-L1 by TNBC cell lines [26,27,28,29,30,34]. Thus, by using luminal-A cell lines that did not express PD-L1 endogenously, we could demonstrate, in an all-or-none manner, that PD-L1 increased several pro-metastatic tumor cell activities. The study of TNBC cells that express PD-L1 in a constitutive manner enabled us to show that elevated PD-L1 expression levels lead to an increased ability of the cells to exert metastasis-promoting functions. Most importantly, in contrast to the down-regulation approaches, these two cell systems enabled us to reveal the regulation of PD-L1 pro-metastatic functions by PD-1, providing yet another level of novelty to our study.

Under these conditions, we identified S283 as a most crucial residue that mediates PD-L1 activities, autonomous and PD-1-induced. So far, conventional signaling motifs were not identified in PD-L1, but three conserved intracellular domains that control PD-L1 activities were detected at the carboxyl terminus of the murine molecule: RMLDVEKC, DTSSK and QFEET [79,80]. In murine PD-L1, the RMLDVEKC motif was required for resistance to interferon-induced apoptosis, whereas the DTSSK motif increased the anti-apoptotic functions of interferons [79,80].

The S283 residue we identified in the current study is located in the same region of PD-L1, between the DTNSK and the HLEET human corresponding sequences (RMMDVKKCGIQDTNSKKQ**S^283^**DTHLEET). Our current research is the first to pinpoint the active pro-metastatic domain of PD-L1 to one specific residue, and to show that the S283 amino acid is essential for exerting tumor-autonomous and PD-1-induced pro-metastatic functions of PD-L1. Our study characterizes S283 as a major regulator of tumor cell growth, release of pro-metastatic mediators and invasion in vitro, and, most importantly, also as a key determinant of tumor growth and metastasis in vivo. By using the xenograft model in which human breast tumor cells were introduced to T cell-deficient mice, we demonstrated that the tumor-intrinsic activities of PD-L1 are cardinal for the ability of the cancer cells to form primary tumors and metastasize, and that they depend on the integrity of the S283 residue located in the intracellular domain of human PD-L1. 

These findings emphasize the high significance of the S283 residue in keeping the functional integrity of PD-L1, joining a recently published study demonstrating that the S283 residue of PD-L1 regulates PD-L1 degradation under conditions of glucose starvation [81]. The involvement of AMP-activated protein kinase (AMPK) in regulating PD-L1 degradation and S283 phosphorylation [81,82] opens the possibility that S283 phosphorylation by AMPK is also involved in down-stream signaling by PD-L1 in cancer cells, and in mediating its cell autonomous pro-metastatic activities. Our modeling of AMPK with a WT-PD-L1-derived peptide that contains S283 and of S283A-PD-L1-derived peptide that contains A283 demonstrated that the latter possessed lower binding efficacy to the catalytic domain of AMPK. To follow up on these findings, in future analyses, we will determine possible mechanisms that may contribute to impaired AMPK-PD-L1 interactions due to S283 modification to A283, possibly leading to modified signaling abilities. 

The findings of our study also shed light on intracellular signaling cascades that mediate tumor cell intrinsic functions of PD-L1. Similar to other investigations that demonstrated roles for the mTOR pathway in mediating PD-L1 activities [83,84], we also noted the activation of mTOR in our system (data not shown); however, our experiments using general inhibitors of two large receptor families that contribute much to tumor progression—GPCRs that signal via Gαi and Ras-activating receptors—substantiated the roles of previously unidentified pathways in controlling the tumor-intrinsic activities of PD-L1 in breast tumor cells. Moreover, our data indicate that each of the two pathways prominently contributes to the autonomous activities of PD-L1: one pathway does not compensate for the absence of the other and blocking both of them simultaneously leads to complete inhibition of pro-metastatic activities.

By addressing specific chemokine receptors that signal via Gαi and mediate tumor cell invasion, we demonstrated that the intrinsic activities of PD-L1 were mediated by the chemokine receptors CXCR1/2, CCR2 and CCR5. Inhibition of these receptors led to potent down-regulation of invasion of WT-PD-L1-MDA cells and, in parallel, also to reduced production of their corresponding ligands (CXCL8, CCL2 and CCL5). In both aspects, the fact that inhibition of one receptor was not complemented by the other receptors indicates that all these receptors act jointly and that their activities are complementary. This possibility is highly supported by the fact that joint inhibition of CXCR1/2, CCR2 and CCR5 led to complete inhibition of tumor cell invasion and of CCL2 and CCL5 production. In this context, it is interesting to note that the antagonists, of all three receptors, exhibited a lower inhibitory effect on CXCL8 expression than on CCL2 and CCL5 expression. These findings suggest that, in parallel to CXCL8, other ELR+ CXC chemokines are involved in the circuits mediated by CXCR1/2, in the regulation of WT-PD-L1-induced invasion of MDA cells. 

The above results reveal that the cell-autonomous activities of PD-L1 lead—primarily via activation of CCR5 and CCR2, and less by triggering CXCR1/2-mediated signals—to increased production of pro-metastatic chemokines, particularly CCL2 and CCL5, but also partly CXCL8. The connection between PD-L1 and chemokine axes was also demonstrated by the TCGA dataset analyses that we performed, demonstrating very high correlation between high PD-L1 expression levels in basal patients and the CCL5-CCR5 and CCL2-CCR2 axes, and less with the CXCL8-CXCR1/2 axis. Specifically regarding CCL2, a recent study demonstrated that transglutaminase 2 has induced resistance to PD-L1/PD-1 inhibition in TNBC by leading to CCL2 production, showing a connection between the PD-L1 and the CCL2 pathways [85]. Moreover, it is possible that PD-L1 activities are connected to chemokine-related pathways also in other aspects, as evidenced by the association of PD-L1 with the chemokine CXCL9 in basal patients [86] and by the cooperativity between CXCR4 (CXCL12 receptor) and PD-L1 inhibitors in pancreatic cancer [87]. 

Overall, these findings indicate that PD-L1 signaling in TNBC cells is channeled in an autocrine manner through the chemokine receptors CXCR1/2, CCR2 and CCR5 to Gαi-mediated signaling events which lead to increased production or release of the key chemokines—CXCL8, CCL2 and CCL5—that then activate these same receptors. As the three chemokines and their receptors were already assumed to promote tumor cell invasion, we suggest that, when the extracellular levels of the chemokines are elevated in WT-PD-L1-expressing cells, they activate the motility apparatus of the cancer cells, leading to potent tumor cell invasion. Thus, these findings demonstrate that autocrine circuits of chemokines and their receptors are essential mediators of the cell-autonomous activities of WT-PD-L1-MDA cells leading to increased invasion. 

## 4. Materials and Methods

### 4.1. Cell Growth and Exposure to PD-1

Human BT-549 (BT) and MDA-MB-231 (MDA) TNBC cells (both from ATCC) were grown in DMEM and RPMI-1640 media, respectively. Human luminal-A MCF-7 cells (from ATCC) and T47D cells (generated and provided by Dr. Keydar [88]) were grown in DMEM medium. Media were supplemented with 10% fetal bovine serum (FBS), 2% L-glutamine and 1% penicillin-streptomycin-amphotericin solution (all from Biological Industries, Beit Ha’emek, Israel). 

To determine PD-1 effects on the cells, a recombinant human PD-1-IgG1 Fc chimera protein (endotoxin-free; #1086-PD; R&D Systems, Minneapolis, MN, USA) was used at 2 μg/mL at time points indicated below. Recombinant human IgG1-Fc was used as control (ctrl) under similar conditions (#110-HG; R&D Systems). PD-1 concentration was selected on the basis of titration experiments (data not shown).

### 4.2. Generation of Cells Over-Expressing WT-PD-L1 or S283A-PD-L1

TNBC and luminal-A cells that over-express human WT-PD-L1 were generated in parallel to control cells (CTRL-vector cells) undergoing a similar procedure with the sham vector, in the following manner: First, to enable intravital in vivo analyses and in vitro studies that may require fluorescent analyses, all cells were infected to express the mCherry-pQCXIN plasmid (with a neomycin selection marker). Then, to generate cells over-expressing WT-PD-L1 (namely, WT-PD-L1-BT, WT-PD-L1-MDA, WT-PD-L1-MCF-7 and WT-PD-L1-T47D cells), retroviral infections with WT-PD-L1-pQCXIP (carrying a puromycin selection marker) were performed in the mCherry-expressing cells. Control cells, termed herein CTRL-vector cells, were infected with an empty pQCXIP vector. To reach this infection stage, HEK-293 cells expressing T-antigen were co-transfected by calcium chloride with a combination of 10 μg of each of the vectors, 7.5 μg helper plasmid encoding pCMVΔR8.2 and 2.5 μg helper plasmid encoding vesicular stomatitis virus-G (VSV-G) proteins. Supernatants were collected after 48 h, filtered through a 0.45 μm mesh, and incubated with the cancer cells in the presence of 8 mg/mL polybrene for 6 h. Seventy-two h following the infection process, the cells underwent selection with 1 μg/mL puromycin (Cat# P-1033; AG Scientific, San Diego, CA, USA) for 2 days. 

In parallel, we established cell systems that would enable us to compare between WT-PD-L1 and S283A-PD-L1. In view of the fact that MDA cells endogenously express PD-L1, we first knocked-out PD-L1 in MDA cells using the Alt-R CRISPR-Cas9 system (Integrated DNA Technologies, Coralville, IA, USA), containing Cas9 nucleases and gRNA targeting PD-L1 (ACCCCAAGGCCGAAGTCATC) or gRNA targeting GFP as a control. Cell clones were screened for the lack of cell surface expression of PD-L1; then, selected clones were sequenced and 3 of them were validated for deletions that led to an “out-of-frame” sequence (Figure S5). These 3 clones were pooled and were infected by the control vector (thus named “KO-PD-L1 cells”) or by the WT-PD-L1/S283A-PD-L1 vectors, which were generated as detailed below.

To generate the WT-PD-L1 construct, total RNA was extracted from MDA-MB-231 TNBC cells and RT reaction was performed. The over-expression construct of human WT-*PD-L1* (NM_014143) was created by PCR amplification of the above cDNA, using the PDL1(81)-Age1-sense primer TATCCAACCGGTCTGTCCGCCTGCAGGGCATT and the PDL1(1020)-Pac1-anti-sense primer CCTGAGTTAATTAAGAGAATCCCTGCTTGAAGATCA. To generate S283A-PD-L1, we used the same sense primer and the anti-sense primer PDL1(S283)-Pac1-GTAGTATTAATTAAGGATTACGTCTCCTCCAAATGTGTATCAGCTTGC. The generated fragments were digested with Age1 and Pac1 and ligated into the corresponding sites of pQCXIP vector (for WT-PD-L1; https://www.addgene.org/vector-database/3870/; accessed on 12 December 2021) and pQCXIH (for S283A-PD-L1; https://www.addgene.org/vector-database/3868/; accessed on 12 December 2021) (Clontech, Santa Clara, CA, USA). PCR products of PD-L1 were sequenced and found to be identical to the published sequence (except for the site of the S283A mutation in S283A-PD-L1). 

Then, MDA cells that were knocked-out for PD-L1 expression (as described above) were infected with the WT-PD-L1 pQCXIP and S283A-PD-L1 pQCXIH vectors and PD-L1 expression was validated by flow cytometry analyses. Corresponding cells, in which PD-L1 was knocked out, were infected by the sham vectors, pQCXIP and pQCXIH. These latter cells were used as controls; to prevent confusion with the CTRL-vector cells mentioned in the previous paragraph, they were called herein “KO-PD-L1-MDA cells”. 

Cell systems of MCF-7 and T47D cells that express WT-PD-L1 and S283A-PD-L1 were also established. Here, the cells did not express endogenous PD-L1 and the mCherry-expressing WT-PD-L1-MCF-7 and WT-PD-L1-T47D cells were the same as those mentioned in the first paragraph of this section. In parallel, mCherry-expressing MCF-7 and T47D cells were infected to express the S283A-PD-L1 pQCXIH vector, generating S283A-PD-L1-MCF-7 and S283A-PD-L1-T47D cells, respectively. Cells infected by the sham pQCXIH vector were also generated, as described above. 

Flow cytometry analyses validated the cell surface expression of PD-L1 by WT-PD-L1-BT, WT-PD-L1-MDA, WT-PD-L1-MCF-7, WT-PD-L1-T47D, S283A-PD-L1-MDA, S283A-PD-L1-MCF-7 and S283A-PD-L1-T47D cells; they also demonstrated that CTRL-vector-MCF-7, CTRL-vector-T47D and KO-PD-L1-MDA cells did not express PD-L1 and that the CTRL-vector-BT and CTRL-vector-MDA cells expressed endogenous PD-L1 levels that were lower than the expression levels of PD-L1 by WT-PD-L1-BT and WT-PD-L1-MDA cells, respectively. 

### 4.3. Flow Cytometry Analyses of PD-L1 Expression

Cell surface expression of PD-L1 was determined by flow cytometry using APC-conjugated anti-human PD-L1 IgG1 antibody #14-5983-82 (Thermo Fisher Scientific, Waltham, MA, USA), followed by FITC-conjugated #115-095-003 (Jackson ImmunoResearch Laboratories, West Grove, PA, USA). Baseline staining was determined by non-relevant isotype-matched control antibodies (#400102, Biolegend). Fluorescence was determined by Flow Cytometer S100EXi (Stratedigm, San Jose, CA, USA), using CELLCAPTURE software (Stratedigm) and analyzed by FLOWJO V10 (BD biosciences, Franklin Lakes, NJ, USA).

### 4.4. Determination of Cancer Cell Growth

Comparisons of growth rates of different cells were performed by plating the cells at equal numbers; four and five days after cell plating, cell numbers were determined by trypan blue exclusion assay in ≥2 replicates/cell type. When exposure to PD-1 was introduced, PD-1 or its ctrl were added to cell cultures at 2 μg/mL one day after cell culturing and cell counts were performed 72 and 96 h later (namely: four and five days after plating). 

### 4.5. ELISA Assays

Cell supernatants were collected from cancer cells that were grown for 48–72 h. When indicated, exposure to PD-1 or its ctrl was carried out for 72 h (added one day after tumor cell plating), followed by collection of cell supernatants. CXCL8, CCL2, CCL5, GM-CSF and soluble ICAM-1 (sICAM-1) levels were determined in cleared supernatants (by centrifugation) and ELISA analyses were performed, using the following antibodies and recombinant proteins: CXCL8: Coating antibodies #500-P28 (Peprotech, Rocky Hill, NJ, USA); Detecting antibodies #500-P28BT (Peprotech); rhCXCL8 standard protein #200-08 (Peprotech). CCL2: Coating antibodies #500-M71 (Peprotech); Detecting antibodies #500-P34BT (Peprotech); rhCCL2 standard protein #300-04 (Peprotech). CCL5: Coating antibodies #500-M75 (Peprotech); Detecting antibodies #BAF278 (R&D Systems); rhCCL5 standard protein #300-06 (Peprotech). GM-CSF: #900-K30 kit (Peprotech). sICAM-1: #900-M464 kit (Peprotech). 

When indicated, WT-PD-L1-MDA cells were cultured and after they adhered to culture plates (after ~4 h) different inhibitors or their DMSO vehicle control were supplemented for 48 h. Then, the levels of the soluble proteins were determined in cleared supernatants by ELISA.

In ELISA analyses of CXCL8, CCL2 and CCL5, HRP-conjugated Streptavidin (#016-030-084, Jackson Immunoresearch laboratories, West Grove, PA, USA) and substrate TMB/E solution (#ES001, Millipore, Burlington, MA, USA) were added, the reaction was stopped by addition of 0.18 M H_2_SO_4_ and absorbance was measured at 450 nm. In the case of GM-CSF and sICAM-1, ABTS substrate was used (#EBK-ABTS, Peprotech) and absorbance was measured at 405 nm. 

### 4.6. Transwell Invasion Assays

To determine cell invasion, the cancer cells were plated in transwells with 8 μm-pore membranes (#3422, Sigma-Aldrich, St. Louis, MO, USA) coated by 20 μg/mL matrigel (#7058006, Sigma-Aldrich). The bottom wells contained medium supplemented with 10% FBS. Migration was performed for the following time points: BT cells: 8 h; MDA cells: 11 h; MCF-7 cells: 21.5 h. When tumor cell invasion through human ECM was performed, BT and MDA cell invasion was carried out through lung ECM (6 mg/mL, MTSLG101, Xylyx Bio, Brooklyn, NY, USA) for 30 h; MCF-7 cell invasion was performed through bone ECM (6 mg/mL, MTSBN101, Xylyx Bio) for 54 h.

In other assays, the invasion of cells grown in the presence of PD-1 or its ctrl was performed; exposure to PD-1 or its ctrl was started one day after cell plating and was carried out for 72 h. Of note, to allow the appropriate density of the invading cells on the membranes, the cells were plated in the transwells in lower concentrations than those mentioned in the first paragraph of this section. When indicated, WT-PD-L1-MDA cells were treated by different inhibitors, as described in the Section 4.5. The cells were then removed from plates and were used in transwell invasion assays, as described above. 

In all invasion experiments, cells that migrated to the lower part of the transwells were fixed, stained with Hemacolor (#111661; Merck, Kenilworth, NJ, USA), photographed using light microscopy and counted at multiple high-power fields (HPF).

### 4.7. Inhibitors: Titration and Selected Concentrations

To determine the effects of the inhibitors on tumor cell viability, they were added to cancer cells for the same time periods used in ELISA assays and transwell invasion experiments (described above). Then, the cells were counted by trypan blue exclusion assay in ≥2 replicates/treatment, as described above. The selected concentrations of all inhibitors did not affect tumor cell viability. The only exception was the CCR2 inhibitor (see below), causing ~40% cell death in the lowest concentration possible. Therefore, in all ELISA assays performed with inhibitors, data were presented after normalization of protein amounts to cell numbers. 

The following inhibitors were used in the study: (1) i-Gαi = Gαi inhibitor—Pertussis Toxin (PTx; 0.1–0.2 µg/mL; #516560, Merck); (2) i-Ras = Ras inhibitor—farnesylthiosalicyclic acid (FTS, Salirasib; 7 µM; #SML1166, Sigma-Aldrich); (3) i-CXCR1/2 = CXCR1/2 inhibitor—Reparixin (30–40 µM; #A12383, ADOOQ Bioscience, Irvine, CA, USA); (4) i-CCR2 = CCR2 inhibitor—CAS 445479-97-0 (0.02 µM; #227016, Merck); (5) i-CCR5 = CCR5 inhibitor—Maraviroc (30 µM; #14641, Cayman Chemical, Ann Arbor, MI, USA). All inhibitors were dissolved in DMSO; thus, this vehicle was used as control in all assays using the inhibitors.

### 4.8. TCGA Dataset Analyses

The TCGA dataset [89] was used in order to perform RNAseq-based gene expression analyses of basal breast cancer patients. The basal subtype was defined based on the PAM50 annotation file provided within the dataset and included 141 patients. Correlation coefficients and *p*-values were analyzed using Pearson’s rho and Spearman rank rho. Values of *p* ≤ 0.05 were considered statistically significant.

### 4.9. Modeling of PD-L1-Peptide Binding to AMPK

Following the study of Yan Y. et al. [90], chains A of 1QMZ and 4RER (the catalytic domain of CDK2 and the catalytic domain of AMPK subunit α, respectively) were aligned by PyMol with RMSD of 1.69Å (Schrödinger & DeLano, 2020; http://www.pymol.org/pymol (accessed on 3 February 2022). Then a CDK2 substrate [91], ATP and Mg^2+^ cation of CDK2 were copied to the structure of AMPK subunit α (only the relevant residues, 11–286, were saved). In PyMol, the CDK2 substrate was mutated twice (‘HHASPRK’ → ‘KKQSDTH’ for WT-PD-L1; ‘HHASPRK’ → ‘KKQADTH’ for S283A-PD-L1), and in Schrödinger the complexes were prepared by ‘Protein Preparation Wizard’ (pH 7 ± 1). MMGBSA calculations (Schrödinger & DeLano) were executed by Prime to each complex. 

### 4.10. In Vivo Studies

Two experimental repeats were performed, each containing the following three groups of mice: (Group 1) Mice injected with KO-PD-L1-MDA cells (*n* = 10 mice in both experiments together); (Group 2) Mice injected with WT-PD-L1-MDA cells (*n* = 9 mice in both experiments together); (Group 3) Mice injected with S283A-PD-L1-MDA cells (*n* = 9 mice in both experiments together). All cell types expressed mCherry in order to enable intravital tumor and metastasis detection by the CRi Maestro imaging system, in intact mice or ex vivo, as required.

Each of the three cell types was mixed with matrigel (#354234, Sigma-Aldrich) at 1:1 ratio; the different cell types were administered at a similar concentration orthotopically to the mammary fat pads of female athymic nude mice (#NUDE242; Envigo RMS, Jerusalem, Israel). Tumor volumes were determined every 3–4 days by caliper. In parallel, mCherry signals at injection site were determined by intravital imaging along the process of tumor growth. 

The three mice groups had different lag periods prior to appearance of primary tumors; thus, each group was sacrificed when tumor sizes reached the limits dictated by the regulations of the Ethics Committee for Animal Use. At termination day, the dimensions of excised tumors were determined in order to calculate their volumes. In parallel, at day of termination, the metastatic load was determined, first by intravital imaging of mCherry signals in the entire mice and then, ex vivo, on excised organs: lymph nodes that were adjacent to the primary tumors, lungs, liver and femur. 

Statistical analyses of kinetics of tumor appearance were performed by Log Rank. Comparisons between tumor volumes were performed by Kruskal–Wallis test, with correction for multiple testing by two-stage linear step-up procedure of Benjamini, Krieger and Yekutieli. The proportions of mice bearing metastases were compared by Chi square test for trend. 

Procedures involving experimental animals were approved by the Tel Aviv University Ethics Committee for Animal Use (Approval no. 04-21-005) and were performed in compliance with local animal welfare laws, guidelines and policies.

### 4.11. Statistical Analyses

Statistical analyses of in vitro studies were performed by two-tailed unpaired Student’s *t*-tests. Statistical analyses of TCGA and in vivo studies were described in the relevant sections. *p* < 0.05 was considered statistically significant.

## 5. Conclusions

Overall, our data emphasize the major importance of tumor cell-autonomous PD-L1 activities in breast cancer. Due to their high mutation rate, which may lead to increased abundance of neo-antigens, it was expected that TNBC patients would demonstrate a considerable response to immunotherapies targeting inhibitory immune checkpoints, such as partners of the PD-L1/PD-1 axis. However, the relatively low success rate of anti-PD-L1/PD-1 treatments in TNBC suggests that mechanisms that are not related to immune suppression were not affected by such ICBs and that other tumor-promoting events remain active at the course of immunotherapy and support tumor progression. 

The findings of our study propose that PD-L1 tumor-intrinsic activities contribute much to the fact that TNBC patients do not respond well to ICB treatments that target the PD-L1/PD-1 axis. Obviously, in patients whose tumors express PD-L1, antibodies to PD-1 do not block the cell-autonomous functions of PD-L1; in parallel, ICBs directed to PD-L1 may have limited abilities to optimally target the immune suppression, as well as the intrinsic metastasis-supporting activities of PD-L1, simultaneously. 

These findings raise the possibility that TNBC patients whose tumor cells express PD-L1 may benefit from combination therapies that target simultaneously the immune-inhibiting and the tumor cell-autonomous checkpoints. Such a therapeutic strategy may combine ICBs directed to PD-1 or CTLA-4—which will reduce immune suppression—alongside with ICBs directed to PD-L1, enabling the latter to have better efficacy in preventing tumor progression, by targeting in full power the intrinsic metastasis-promoting functions of PD-L1. By establishing such combined therapeutic approaches, it is possible that better treatment options could be offered to patients experiencing the most aggressive disease type of breast cancer.

## Data Availability

The data presented in this study are available on request from the corresponding author.

## Supplementary Materials

The following supporting information can be downloaded at: https://www.mdpi.com/2072-6694/14/4/1042/s1, Figure S1: Autonomous activities of PD-L1 increase tumor cell invasion through ECM substrates, and these activities are further potentiated by exposure to PD-1; Figure S2: PD-L1 expression is highly correlated with the CCL2-CCR2 and the CCL5-CCR5 axes in basal breast cancer patients; Figure S3: Invasion through ECM substrates—via cell-autonomous and PD-1-induced activities of PD-L1-are fully dependent on S283 integrity; Figure S4: Modeling of AMPK binding to peptides containing S283 and S283A; Figure S5: PD-L1 sequences targeted for generation of PD-L1 knock-out by CRISPR Cas9; Table S1: MMGBSA calculations of binding efficiency of WT-PD-L1-derived peptide and S283A-PD-L1-derived peptide to AMPK.
