# Correction: Erlichman et al. Tumor Cell-Autonomous Pro-Metastatic Activities of PD-L1 in Human Breast Cancer Are Mediated by PD-L1-S283 and Chemokine Axes. *Cancers* 2022, *14*, 1042

**DOI:** 10.3390/cancers14112579

**Published:** 2022-05-24

**Authors:** Nofar Erlichman, Tamir Baram, Tsipi Meshel, Dina Morein, Benny Da’adoosh, Adit Ben-Baruch

**Affiliations:** 1 The Shmunis School of Biomedicine and Cancer Research, George S. Wise Faculty of Life Sciences, Tel Aviv University, Tel Aviv 69978-01, Israel; nofarerlichman@gmail.com (N.E.); tamiros21@gmail.com (T.B.); tsipi.meshel@gmail.com (T.M.); dinamorein1@gmail.com (D.M.); 2 Blavatnik Center for Drug Discovery, Tel Aviv University, Tel Aviv 69978-01, Israel; daadoosh@tauex.tau.ac.il

The authors apologize for several typos/technical inaccuracies in the original article [1]:

(1) References 36 and 38 have mistakenly addressed sPD-L1, instead of sPD-1; relevant references on sPD-1 are: PMIDs 33499013, and 26859684 (Pubmed). In page 19, line 33 from the top, the correct reference is 33.
(2) In flow cytometry, the antibodies used for PD-L1 were “#14-5983-82 (Thermo Fisher Scientific, Waltham, MA, USA), followed by FITC-conjugated #115-095-003 (Jackson ImmunoResearch Laboratories, West Grove, PA, USA)”.
(3) Legend to Figure 4 got corrupted and should be: “The cell-autonomous activities of WT-PD-L1 act via chemokine receptors to upregulate the expression of their corresponding chemokines”.

The authors apologize for any inconvenience caused and state that the scientific conclusions are unaffected. The original article has been updated.
