# Supplementary material for: Tumor Cell-Autonomous Pro-Metastatic Activities of PD-L1 in Human Breast Cancer Are Mediated by PD-L1-S283 and Chemokine Axes
Source: Cancers (Basel). 2022 Feb 18;14(4):1042. doi: 10.3390/cancers14041042 (PMC8870053; doi:10.3390/cancers14041042)
Supplement: Supplementary file 1 [file cancers-14-01042-s001.zip › cancers-1550736-supplementary.pdf]

Fig. S1

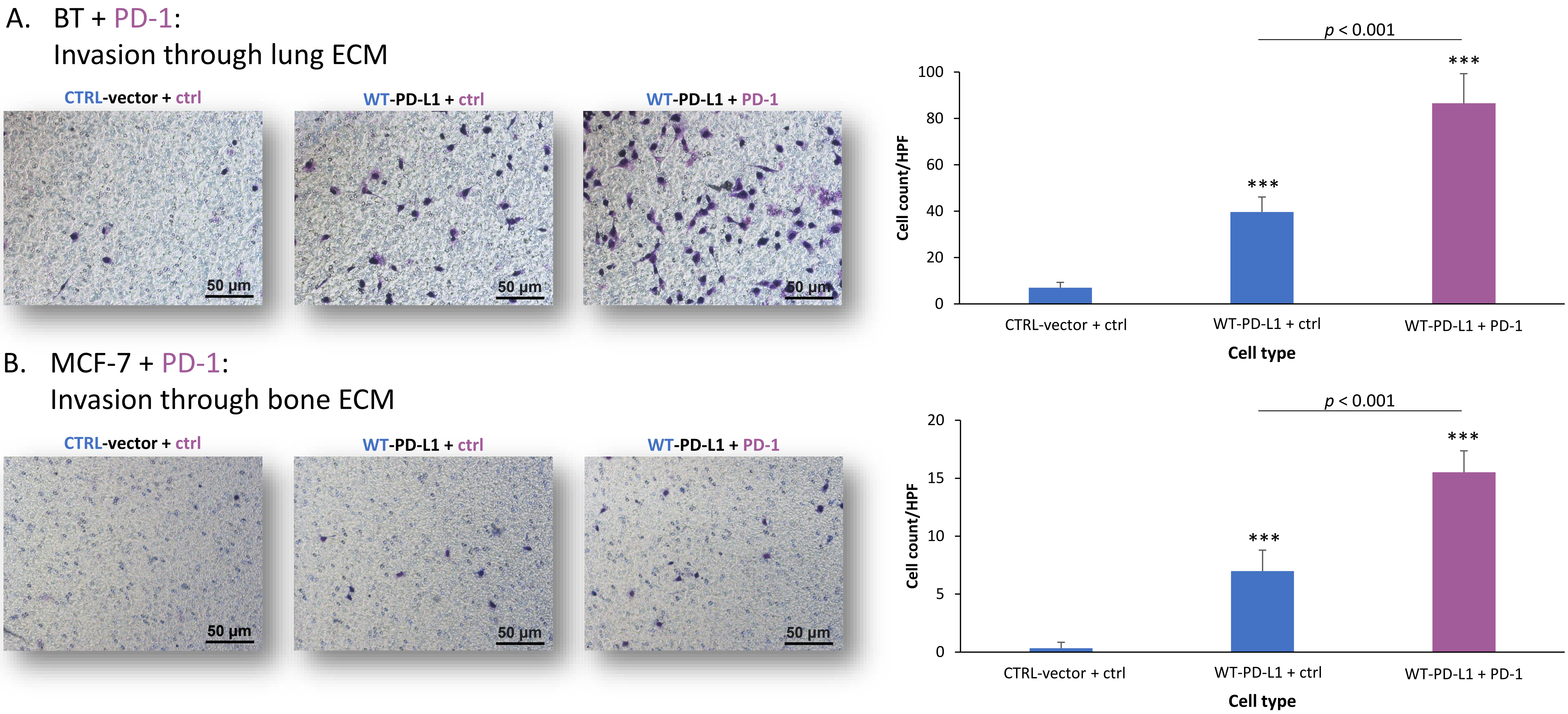

Autonomous activities of PD-L1 increase tumor cell invasion through ECM substrates, and these activities are further potentiated by exposure to PD-1
Invasion of **(A)** WT-PD-L1-BT cells and **(B)** WT-PD-L1-MCF-7 cells was determined in ECM-coated transwells. WT-PD-L1-over-expressing cells and CTRL-vector cells were cultured and 24 hrs later, PD-1 or its control (ctrl), were added at 2μg/ml for 72 hrs. Tumor cell invasion was performed under the following conditions: BT cells: Lung ECM, for 30 hrs; MCF-7 cells: Bone ECM, for 54 hrs. Each panel demonstrates the results of a representative experiment out of n=3. ***p<0.001.

Fig. S2

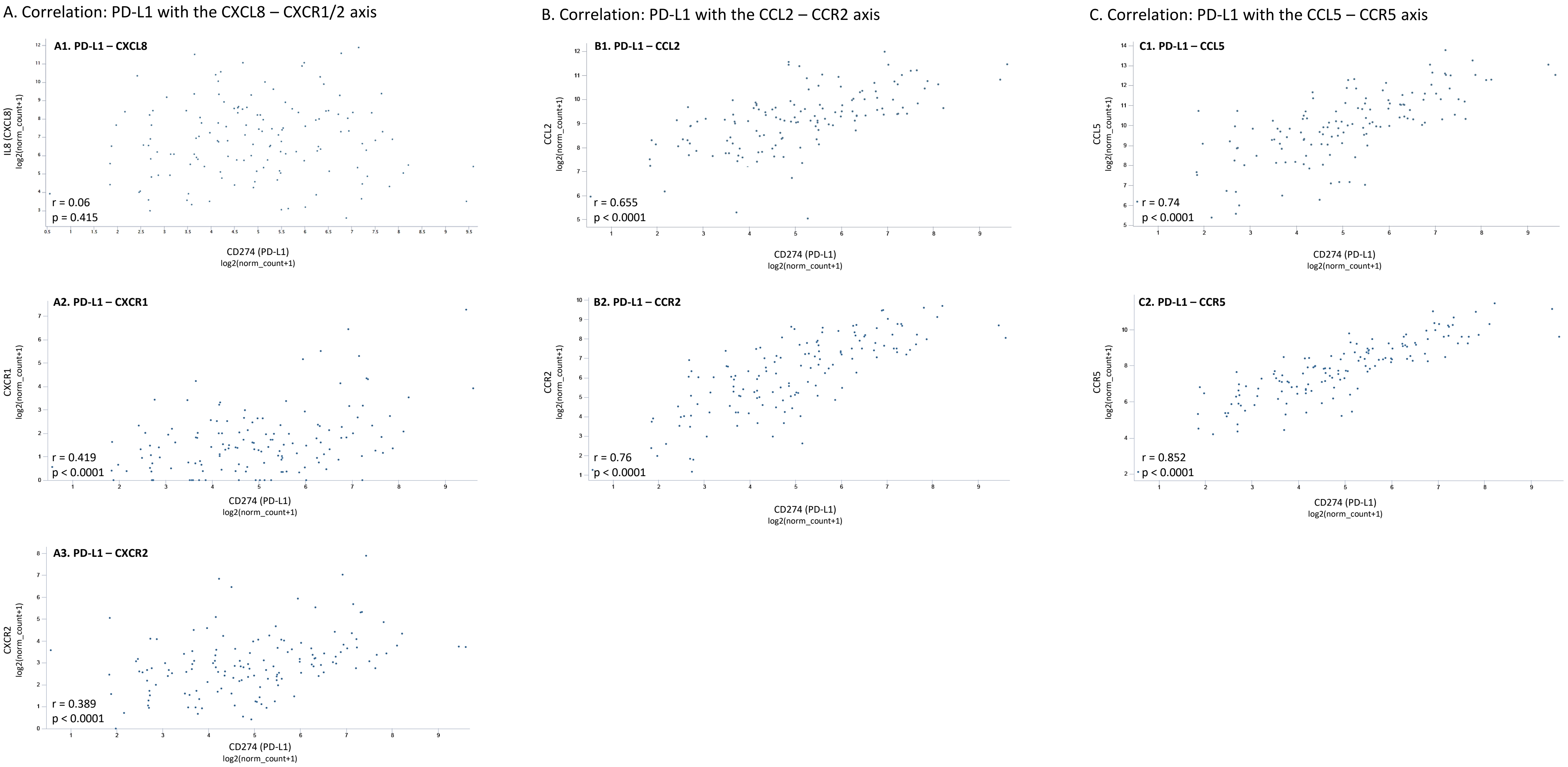

PD-L1 expression is highly correlated with the CCL2 - CCR2 and the CCL5 - CCR5 axes in basal breast cancer patients

Data available in the TCGA dataset was used to identify correlations in gene expression in basal patients. n=141. **(A)** Correlations of PD-L1 with members of the CXCL8 – CXCR1/2 axis. (A1) CXCL8, (A2) CXCR1, (A3) CXCR2. **(B)** Correlations of PD-L1 with members of the CCL2 – CCR2 axis. (B1) CCL2, (B2) CCR2. **(C)** Correlations of PD-L1 with members of the CCL5 – CCR5 axis with PD-L1. (C1) CCL5, (C2) CCR5.

Fig. S3

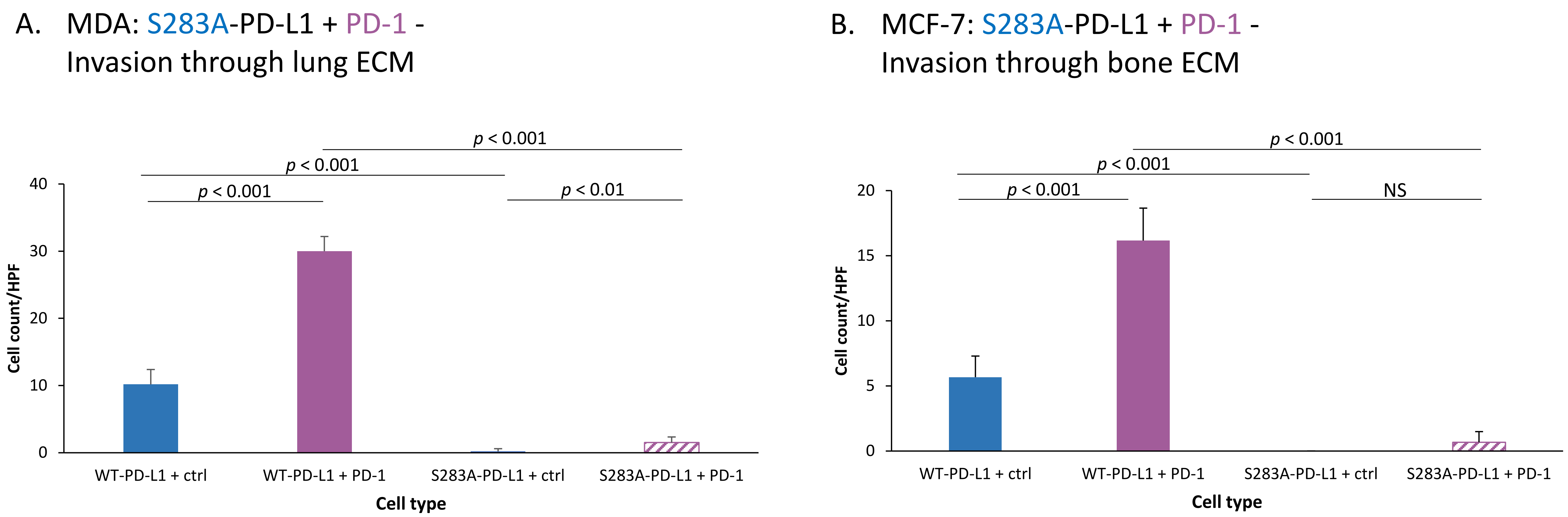

Invasion through ECM substrates - *via* cell-autonomous and PD-1-induced activities of PD-L1 - are fully dependent on S283 integrity

Invasion of **(A)** MDA cells and **(B)** MCF-7 cells that over-expressed WT-PD-L1 or S283A-PD-L1 was determined in transwell assays, after exposure to PD-1 or its control (ctrl). Cell invasion was determined in ECM-coated transwells for the time points indicated in Figure S1: lung ECM in the case of MDA cells and bone ECM in the case of MCF-7 cells. PD-1 or its control ctrl were added one day after culturing, at 2μg/ml, for 72 hrs. Each panel demonstrates the results of a representative experiment out of n=3. p values are demonstrated in the Figure. NS, Non-significant.

Fig. S4

A. AMPK structure

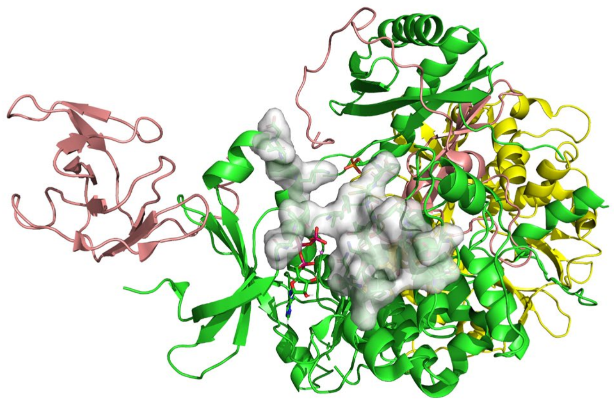

B1. AMPK – S283 (in WT-PD-L1)

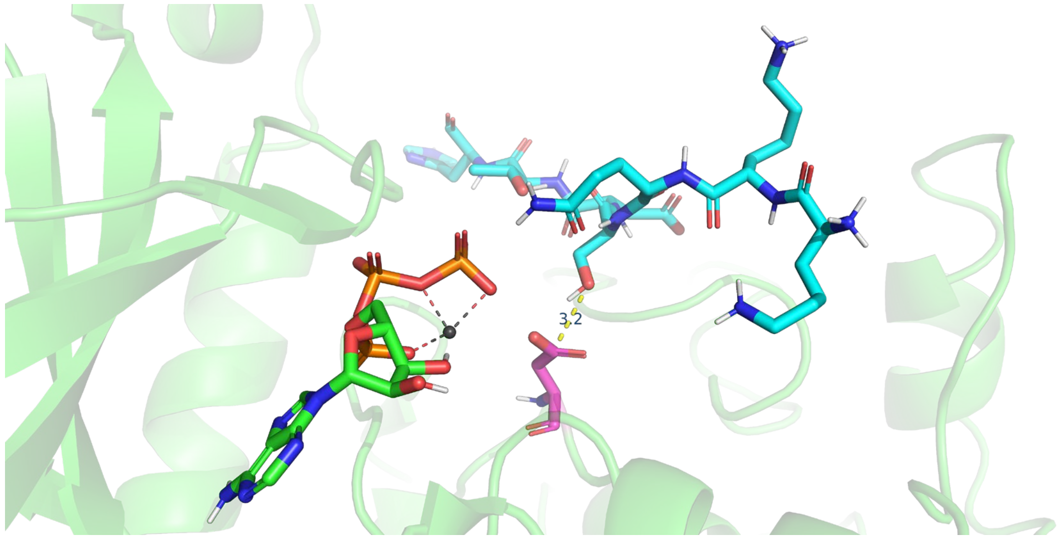

B2. AMPK – A283 (in S283A-PD-L1)

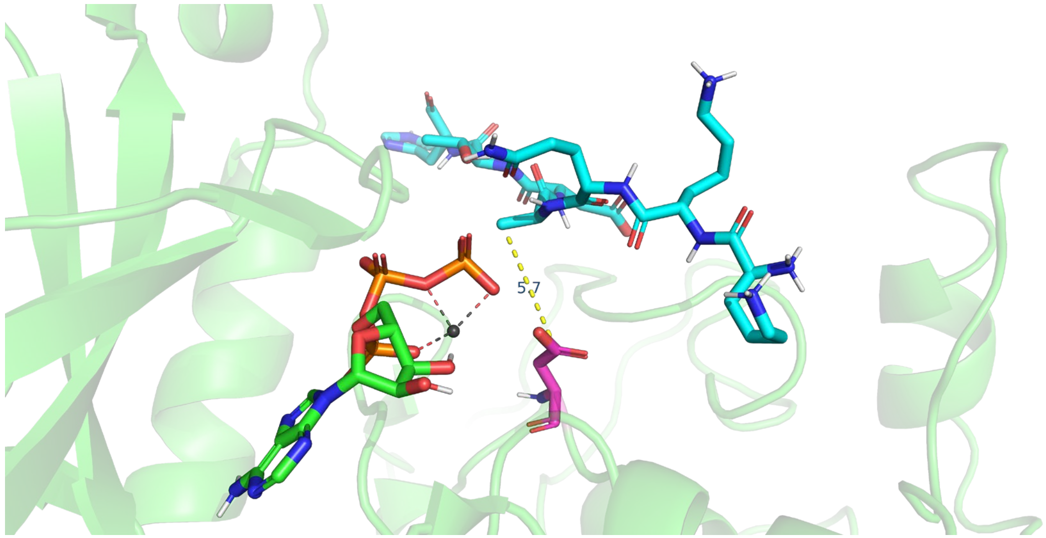

Modeling of AMPK binding to peptides containing S283 and S283A

(A) The structure of AMPK, determined by PDB ID 4RER. Green, the catalytic α subunit of AMPK. White, surface residues that make contacts with substrates. Burgundy, the β subunit of AMPK. Yellow, the γ subunit of AMPK. **(B)** Modeling of the 7-aa peptide complexes that contain either the native S283 residue that is expressed in WT-PD-L1 (B1) or the A283 residue of mutated S283A-PD-L1 (B2), in AMPK catalytic site. Figures were generated by PyMol. Non polar hydrogens are not demonstrated. Green, carbons in AMPK. Magenta, catalytic aspartate. Cyan, 7-aa peptides. In B1, the dashed line demonstrates the distance between carbon in aspartate and hydrogen in serine. In B2, due to the lack of hydrogen in alanine, the dashed line demonstrates the distance between the carbon in aspartate and carbon in alanine.

Fig. S5

CRISPR Cas9 gene editing in MDA cells

Identification of clones that do not express surface PD-L1, determined by flow cytometry

Selection of PD-L1-non-expressing clones for sequencing

Sequencing and selection of 3 clones in which deletion has led to an **“out-of-frame”** sequence:

Cell clone 1

```
WT PD-L1       552  CAAGGCCGAAGTCATCTGGACAAGCAGTGACCATCAAGTCCTGAGTGGTAAGACCACCAC  611
                    ||             ||||||||||||||||||||||||||||||||| || |||| ||
After CRISPR   57   CA-------------CTGGACAAGCAGTGACCATCAAGTCCTGAGTGGAAATACCATCAT  103
```

Cell clone 2

```
WT PD-L1       552  CAAGGCCGAAGTCATCTGGACAAGCAGTGACCATCAAGTCCTGAGTGGTAAGACCACCAC  611
                    |||||||||||||                           ||||||||||||||||||||
After CRISPR   58   CAAGGCCGAAGTC---------------------------CTGAGTGGTAAGACCACCAC  90
```

Cell clone 3

```
WT PD-L1       552  CAAGGCCGAAGTCATCTGGACAAGCAGTGACCATCAAGTCCTGAGTGGTAAGACCACCAC  611
                    |||||||||||||                           ||||||||||||||||||||
After CRISPR   57   CAAGGCCGAAGTC---------------------------CTGAGTGGTAAGACCACCAC  89
```

The 3 clones were combined, generating one cellular pool

Infection with:

Control vector = KO-PD-L1 cells

WT-PD-L1 vector = WT-PD-L1 cells

S283A-PD-L1 vector = S283A-PD-L1 cells

PD-L1 sequences targeted for generation of PD-L1 knock-out by CRISPR Cas9

To produce MDA cells in which WT PD-L1 was knocked-out (KO), the CRISPR Cas9 gene editing methodology was used, as described in “Materials and methods”. Following the process demonstrated in the current Figure, flow cytometry has validated correct PD-L1 expression pattern, as shown in Figure 8.

Table S1

	**MMGBSA_dG_Bind**									
Title	total	Coulomb	Covalent	Hbond	Lipo	Packing	SelfCont	Solv_GB	Solv_SA	vdW
Native	-26.75	-17.96	0.04	-3.80	-4.19	0.00	0.00	41.34		-42.18
Mutant	-2.48	-25.24	2.35	-2.13	-5.68	0.00	0.00	77.17		-48.94
	**Lig_Strain**									
Title	total	Coulomb	Covalent	Hbond	Lipo	Packing	SelfCont	Solv_GB	Solv_SA	vdW
Native	11.85	30.40	0.51	2.43	0.02	0.00	0.00	-23.82		2.30
Mutant	12.58	11.00	2.82	0.04	-0.11	0.00	0.00	-3.84		2.66
	**Ligand**									
Title	total	Coulomb	Covalent	Hbond	Lipo	Packing	SelfCont	Solv_GB	Solv_SA	vdW
Native	-242.61	-193.27	33.38	-3.50	-1.98	0.00	0.00	-88.56		11.31
Mutant	-238.04	-163.98	27.32	-1.54	-1.87	0.00	0.00	-110.91		12.94
	**Complex**									
Title	total	Coulomb	Covalent	Hbond	Lipo	Packing	SelfCont	Solv_GB	Solv_SA	vdW
Native	-11394.59	-8251.71	1185.92	-147.01	-606.27	-23.45	-13.31	-2262.25		-1276.51
Mutant	-11370.35	-8230.92	1179.50	-143.57	-607.68	-23.44	-13.31	-2248.63		-1282.29
	**Receptor**									
Title	total	Coulomb	Covalent	Hbond	Lipo	Packing	SelfCont	Solv_GB	Solv_SA	vdW
Native	-11125.23	-8040.48	1152.51	-139.71	-600.10	-23.45	-13.31	-2215.03		-1245.65
Mutant	-11129.83	-8041.70	1149.83	-139.90	-600.14	-23.44	-13.31	-2214.88		-1246.29
	**MMGBSA_dG_Bind(NS)**									
Title	total	Coulomb	Covalent	Hbond	Lipo	Packing	SelfCont	Solv_GB	Solv_SA	vdW
Native	-38.60	-48.37	-0.48	-6.23	-4.21	0.00	0.00	65.16		-44.48
Mutant	-15.06	-36.25	-0.47	-2.17	-5.57	0.00	0.00	81.00		-51.61
	**Rec_Strai**									
Title	Energy	Coulomb	Covalent	Hbond	Lipo	Packing	SelfCont	Solv_GB	Solv_SA	vdW
Native	0.00	0.00	0.00	0.00	0.00	0.00	0.00	0.00		0.00
Mutant	0.00	0.00	0.00	0.00	0.00	0.00	0.00	0.00		0.00
	**Prime_MMGBSA_ligand**									
Title	efficiency	efficiency_sa	efficiency_ln							
Native	-0.46	-0.69	-5.29							
Mutant	-0.04	-0.07	-0.49							

MMGBSA calculations of binding efficiency of WT-PD-L1-derived peptide and S283A-PD-L1-derived peptide to AMPK

The Table demonstrates MMGBSA calculations of relevant parameters. Native: S283-containing WT-PD-L1-derived peptide; Mutant: S283A-PD-L1-derived peptide.
